# Limitations of *p*_50_ as a measure of seed longevity in comparative studies and the way forward

**DOI:** 10.1002/aps3.70032

**Published:** 2025-11-29

**Authors:** Lea Klepka, Angelino Carta, Fiona R. Hay, Anna Bucharova

**Affiliations:** ^1^ Conservation Biology, Department of Biology Philipps University Marburg Karl‐von‐Frisch‐Straße 8 Marburg 35043 Germany; ^2^ Department of Biology, Botany Unit University of Pisa Pisa 56126 Italy; ^3^ Department of Agroecology Aarhus University Forsøgsvej 1 Slagelse 4200 Denmark

**Keywords:** comparative study, *p*_50_, probit analysis, seed longevity, seed viability, σ (sigma), viability loss rate

## Abstract

**Premise:**

Studies comparing seed longevity between species or genotypes commonly measure seed longevity as the time it takes for seed lot viability to drop to 50% (*p*_50_). However, *p*_50_ is influenced by the initial viability. Although standard protocols for comparative studies thus recommend using seed lots with similar and high initial viability (>85%), variation in viability strongly influences *p*_50_.

**Methods:**

We simulated seed viability decline across a range of initial viabilities and rates of probit viability loss (σ) to illustrate how variation in initial viability affects *p*_50_ estimates and to test approaches for reducing this bias.

**Results:**

For hypothetical seed lots with identical rates of seed viability loss, variation in initial viability leads to a threefold variation in *p*_50_ estimates. Narrowing the initial viability (e.g., to 85–95%) reduced this bias. Alternatively, *p*_50_ can be recalculated to a standardized value of initial viability (e.g., 90%), which makes it proportional to the rate of probit viability loss. The most straightforward measure of seed longevity for comparative studies is the probit rate of viability loss itself, represented by σ from the viability equation.

**Conclusions:**

*p*_50_ is confounded by variation in initial seed lot viability and is suboptimal for comparative studies of seed longevity among species or genotypes. Robust measures of seed longevity include the rate of probit viability loss (σ) or *p*_50_ standardized to a certain initial seed viability.

Seed longevity—the period over which seeds remain viable (Nadarajan et al., 2023)—is a critical seed trait. A detailed, comprehensive understanding of how long seeds maintain viability could facilitate effective seed storage in conservation seed banks and gene banks, and enhance understanding of seed persistence in the soil. There is a common understanding that seed longevity strongly depends on environmental conditions pre‐ and post‐harvest as well as during storage (Solberg et al., 2020; Corbineau, 2024) and varies among species (Probert et al., 2009; Merritt et al., 2014), but there is also some variation between accessions of the same species, both for crops and wild species (e.g., Probert et al., 2009; Mondoni et al., 2011; Lee et al., 2019; White et al., 2023; Balasupramaniyam et al., 2025).

Comparative studies of seed longevity among species or populations within species typically aim to predict seed longevity based on phylogeny, seed traits, climate at the seed origin, or other factors (Probert et al., 2009; Mondoni et al., 2011; Merritt et al., 2014). For example, Probert et al. (2009) compared seed longevities across 195 wild species from around the globe and found that endospermic seeds are shorter‐lived than non‐endospermic seeds, and that seeds from hot and dry environments are longer‐lived than seeds from cold and moist environments. Similarly, Merritt et al. (2014) focused on seed longevities of 172 species from across Australia and found weak but significant positive correlations between seed longevity and seed weight, temperature, and precipitation. Mondoni et al. (2011) compared seeds of alpine and lowland populations of the same species and showed that alpine populations were shorter‐lived than lowland ones. These studies are interested in seed longevity as a trait inherent to species, population, or genotype.

To study seed longevity, comparative studies typically expose seeds to specific constant aging conditions, depending on the research questions, with seed survival then monitored through germination tests at predefined intervals (e.g., Walters et al., 2005; Probert et al., 2009; Carta et al., 2018; Moravcová et al., 2022). Studies focusing on longevity of seeds in conservation seed banks commonly follow the comparative longevity protocol developed by the Millennium Seed Bank of the Royal Botanic Gardens Kew (Newton et al., 2014; Royal Botanic Gardens Kew, 2022). In brief, seeds are exposed to 60% relative humidity and a temperature of 45°C or 60°C, conditions that are assumed to accelerate chemical processes similar to the ones that happen in the seeds during long‐term storage in ex situ facilities (Delouche and Baskin, 1973; Rajjou and Debeaujon, 2008). Individual samples are removed from the artificial aging conditions after 1, 2, 5, 9, 20, 30, 50, 75, 100, and 125 days, and the proportion of viable seeds is determined. The proportion of germinated seeds (as a binomial variable) is then related to the time in aging conditions using a probit link function. The probit transformation converts binomial responses onto a linear scale with a normal distribution to fit the seed viability equation (Ellis and Roberts, 1980):

(1) $$v=K_{\text{i}}-p/\sigma$$

Where *v* is seed viability (in probits) of the seed sample after *p* days in the aging environment, *K*_i_ is the *y*‐intercept (i.e., the theoretical initial viability [in probits]), and σ (sigma) is the time for viability to decrease by 1 probit. The σ parameter directly describes the rate of seed viability decay on a probit scale. To interpret *K*_i_ on a more intuitive scale, it can be back‐transformed from probits to percentages, *K*_i_(%), using the inverse of the probit function. *K*_i_, as the theoretical initial viability of a seed lot, estimated based on fitting the survival curve through probit analysis, is similar but likely not identical with the experimentally observed seed viability at the start of the aging experiment.

As a measure of seed longevity, comparative studies commonly use the time when germination upon removal from the aging environment has decreased to 50% of the tested seeds, known as *p*_50_ (Priestley et al., 1985; Walters et al., 2005; Probert et al., 2009; Mondoni et al., 2011; Satyanti et al., 2018). However, *p*_50_ inherently depends on both the rate of viability decline (σ) and the initial viability (*K*_i_):

(2) $$p_{50}=K_{i}\times \sigma$$

When the initial viability is higher (e.g., 95%), it takes longer for seed viability to decrease to 50% compared to when the initial viability is lower (e.g., 85% or 70%) (Figure 1). The initial viability *K*_i_ is a seed lot (accession) trait and can be affected by the age of the seeds at the beginning of the experiment, post‐harvest treatments, or other environmental effects. Thus, any accession of any species could have any reasonable level of initial viability. While the initial viability, estimated from standard germination tests, is a useful indicator of seed quality, comparative studies are instead interested in the rate of seed viability loss, which is specific for species, populations, or genotypes. This rate of seed viability loss is best represented by σ (Equation 2), which is independent of the initial viability. Consequently, Hay et al. (2022) stated that σ would be the most suitable measure of seed longevity in storage. At the same time, the authors acknowledge that σ is not intuitive to understand and recommend using *p*_50_ if all included accessions have similar and high initial seed viability. In that case, *p*_50_ should be proportional to σ (Hay et al., 2019, 2022).

**Figure 1:**
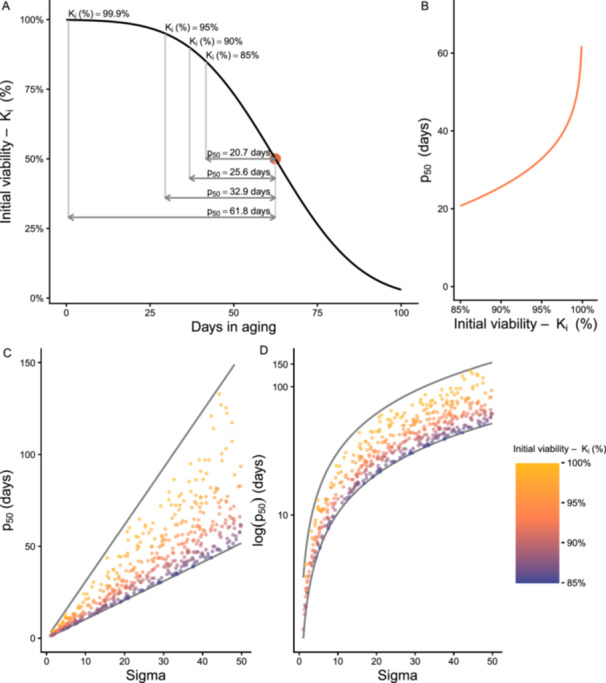
Illustration of how *p*_50_ estimates depend on the initial viability of a seed lot. (A) Seed survival curve describing the decline in viability of hypothetical seed accessions with a rate of probit viability loss of σ = 20 days. (B) *p*_50_ exponentially increases as the initial viability of the seed lot at the start of the aging experiment increases, here illustrated for a hypothetical accession with σ = 20 days. (C) Illustration of the noise introduced to *p*_50_ estimates by using seed accessions with initial viabilities varying between 85% and 99.9%. Points are *p*_50_ estimates for 500 hypothetical seed accessions with σ randomly varying between 1 and 50 days and initial viabilities varying between 85% and 99.9%. (D) The same illustration of introduced noise shown in C, with a logarithmic scale for the *y*‐scale. The gray lines in C and D correspond to *p*_50_ with *K*_i_ fixed to 85% and 99.9%.

To date, the similar and high initial viability recommended by Hay et al. (2022) has not been clearly defined. In practice, most studies have used seeds with an initial viability of at least 80% (Probert et al., 2009; Mondoni et al., 2011; Merritt et al., 2014; but see also Satyanti et al., 2018 who used 75%), while the protocol developed by the Millennium Seed Bank of the Royal Botanic Gardens Kew recommends using seeds with at least 85% initial viability (Royal Botanic Gardens Kew, 2022). This allows for using seeds with initial viabilities ranging between 85% and 100%, which inevitably introduces some unintended variation to the *p*_50_ estimates; in reality, however, a narrower range is likely covered, especially for prestored seed bank accessions. The question is whether the magnitude of this unintended variation is acceptable for comparative studies among accessions.

In this study, we illustrate the magnitude of the unintended variability in *p*_50_ caused by using seed lots that range in initial viability between 85% and 100%. While the results presented below might be obvious for seed physiologists familiar with probit analysis, they were unexpected to the first and last authors of this study—ecologists who only recently started to work on seed longevity. Given that *p*_50_ estimates from seed lots with varying initial viability are commonly used in comparative studies, we believe that illustrating the magnitude of the variability in *p*_50_ will be useful for fully comprehending the consequences of this practice. We then outline three possible solutions for how to reduce or remove the unintended variation in the seed longevity estimates.

## METHODS

All simulations were performed in R Statistical Software (R version 4.4.2; R Core Team, 2025). For clarity of communication, we do not provide equations in the main text (Fawcett and Higginson, 2012), but see, for example, Hay et al. (2014). We provide an annotated R script in Appendix S1 (see Supporting Information with this article), allowing readers to follow each calculation step of the models, replicate the results, and adjust thresholds to suit individual needs.

To achieve our goal, we adopted a three‐step procedure: (1) we illustrated the basic relationship between *p*_50_ and initial viability, *K*_i_(%), and then (2) we illustrated the variation in *p*_50_ estimates across seed accessions that vary in initial seed viability. Finally, (3) we explored possible solutions to reduce or remove the unintended variation in *p*_50_ estimates.

In the first step, we illustrated the basic relationship between *p*_50_ and initial viability, *K*_i_(%), using a hypothetical accession with a fixed rate of probit viability loss (σ = 20 days). This means that seed viability decreases by one probit—for example, from 84.1% to 50%, or from 97.7% to 84.1%—every 20 days. While we kept σ constant, we varied initial seed viability in 0.1% steps between 85% and 99.9% (100% is theoretically impossible). We then calculated *p*_50_ using Equation 2 and plotted it against the initial seed viability.

In the second step, we illustrated the magnitude of unintended variation in *p*_50_ estimates that is introduced by using seed accessions that vary in initial seed viability. For this, we simulated 500 hypothetical seed accessions with σ randomly varying between 1 and 50 days and initial viability between 85% and 99.9%. This example mimics a comparative analysis across many accessions. We calculated *p*_50_ using Equation 2 and then related the obtained *p*_50_ to the σ of the given accession. We further show the relationship between log(*p*_50_) and σ, as *p*_50_ is often log‐transformed in comparative analyses (Probert et al., 2009; Merritt et al., 2014; Satyanti et al., 2018).

In the third step, we explored possible solutions to reduce or remove the unintended variation introduced to *p*_50_ estimates by using seed lots varying in initial viability. As one option, we explored the effect of restricting the range of initial viabilities to 75–85%, 80–90%, 85–95%, or 90–99.9%. To do this, we first simulated 500 hypothetical seed accessions as in the paragraph above, with σ randomly varying between 1 and 50 days and initial viability within the given intervals. As an alternative option, we directly used the slope of probit viability loss, σ, although it might be hard to interpret. To help the reader to more intuitively understand the σ, we visualize the loss of viability that happens in σ days, depending on the initial viability. The last option we explored was to recalculate *p*_50_ based on a standardized value of *K*_i_, e.g., 90% (i.e., as a multiple of σ), so that it is independent of the true initial viability. We then report the adjusted *p*_50_ estimate as the time needed for viability of a seed lot to decrease from 90% to 50%.

## RESULTS

For a hypothetical seed accession with σ = 20 days, the estimates of *p*_50_ varied between 20.7 and 61.8 days when calculated for seed accessions with initial viabilities between 85% and 99.9%, respectively. The differences between *p*_50_ estimates were especially large when using seed lots with viabilities above 95% (Figure 1A, B). Independent of the σ value, the *p*_50_ based on a seed lot with 99.9% initial viability was 2.98 times larger than the *p*_50_ based on a seed lot with 85% initial seed viability, which introduces substantial noise to comparisons across accessions (Figure 1B). While log‐transformation of the *p*_50_ reduced the noise to some extent, *p*_50_ based on a seed lot with 99.9% viability was consistently larger than *p*_50_ based on a seed lot with 85% viability, with an additive difference of 1.1 units on the logarithmic scale (Figure 1C).

**Figure 2:**
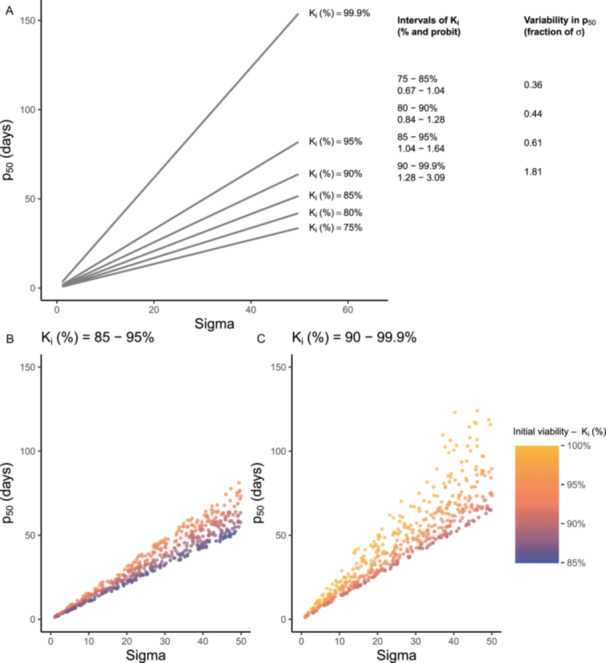
(A) The relationship between *p*_50_ and σ when the initial viability is fixed to a specific value. Note the absence of any variation in *p*_50_. On the right are variations in *p*_50_ estimates when only seed lots from a restricted range of initial viabilities are used. (B) and (C) illustrate the variation in *p*_50_ estimates for 300 hypothetical seed accessions that randomly vary in σ from 1 to 50 days (*x*‐axis) and in initial viability from 85% to 95% (B) or from 90% to 99.9% (C).

Restricting the initial seed viability to a specific range also reduced the noise in the resulting *p*_50_. For example, when we restricted the initial viability to 85–95%, the difference between the highest and lowest possible *p*_50_ at the same σ was only 0.61 σ (i.e., the probit difference between 85% and 95%). In contrast, using seeds with initial viabilities ranging between 90–99.9%, this difference increased to 1.81 σ—almost three times larger (Figure 2). When we fixed the initial seed viability to 90%, or any other value, the *p*_50_ estimate increased proportionally with σ without any noise (Figure 2A).

## DISCUSSION

Comparative studies of seed longevity in storage typically involve exposing seeds to high temperature and humidity for different time periods to accelerate seed aging. As a measure of seed longevity, most studies use the *p*_50_ estimate, which is the time it takes for the viability of the seed lot to decrease to 50% (i.e., half the seeds are able to germinate and half are not able to germinate and presumed dead). The *p*_50_ values are typically estimated using seed lots with high initial viability. However, here we illustrate that using seed lots with >85–100% initial seed viability introduces a threefold difference in the resulting *p*_50_ estimates. To address this issue, we evaluate three potential solutions and alternatives to the *p*_50_ as currently in practice.

Most of the existing large comparative studies on seed longevity in storage have used *p*_50_ estimates based on seed lots with varying initial viability (e.g., Probert et al., 2009; Mondoni et al., 2011; Merritt et al., 2014; Satyanti et al., 2018). These *p*_50_ estimates are likely influenced by substantial variation in initial viability. While using seeds with higher initial viability improves the precision of the individual *p*_50_ estimates, here we show that including seed lots with 95–99.9% viability actually produces the largest variation in *p*_50_. Seed lots with such high viabilities have been commonly included in comparative studies of seed longevity (e.g., White et al., 2023; Balasupramaniyam et al., 2025). Thus, the *p*_50_ estimates in these studies may mask subtler relationships between longevity and other factors, such as seed weight or climatic parameters. Log‐transformation of the *p*_50_ estimates before relating it to environmental parameters transformed the noise from multiplicative to linear along the *p*_50_ values, but the data are still noisy, and the impact of the noise on the results remains unclear.

The *p*_50_ estimates are calculated from the probit seed viability curve, specifically from the intercept (theoretical initial viability) and slope (i.e., the rate of viability loss [σ] on the probit scale) (Equation 2). Because σ is unaffected by *K*_i_, it is fully comparable between seed lots. Unfortunately, the largest comparative studies of seed longevities (Probert et al., 2009; Merritt et al., 2014; Satyanti et al., 2018) do not report initial viabilities or publish underlying data and, thus, it is not possible to calculate σ (but see Lee et al., 2019, 2020). It is therefore impossible to verify whether results of these studies also hold without the noise caused by the variation in initial viabilities. To improve comparability, Hay et al. (2022) called for publishing the theoretical initial viabilities and σ values alongside the *p*_50_ estimates, yet this has been done only by a few authors so far (e.g., Kochanek et al., 2009; Carta et al., 2018; Hay et al., 2019; White et al., 2023; Balasupramaniyam et al., 2025).

In summary, the *p*_50_ estimate is a suboptimal measure of intrinsic seed longevity in storage for comparative studies because it contains substantial noise caused by variation in the initial viabilities of the seed lots at the start of an aging experiment. Below, we suggest a practical approach to reduce the noise in *p*_50_ estimates and present two alternative measures that are not affected by the noise caused by varying initial viability.

### Solution 1: Use seed lots with a limited range of initial viabilities

**Figure 3:**
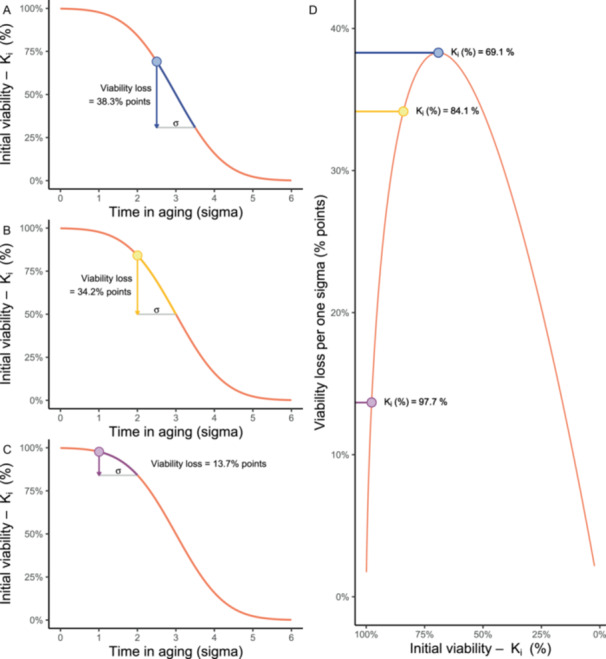
Illustration of loss of seed viability per one σ (i.e., the fixed number of days) and how this loss varies with the initial seed viability. (A–C) Percentage viability loss over one σ on the seed survival curve for three initial viabilities. (D) The magnitude of percentage viability loss per one σ as a function of initial viability.

One way to reduce the unintended variation in *p*_50_ estimates is to restrict the seed accessions that are used for the experiment to a defined range of initial viabilities. This will reduce the number of usable accessions, but also the noise in the resulting *p*_50_ estimates. If set sufficiently high, it will also reduce the likelihood of obtaining negative estimates of *p*_50_, which are also difficult to handle in subsequent analyses. Because this solution makes the fewest changes to the established practice of using *p*_50_ estimates, it might be most acceptable for some researchers. In this context, the least noise will be achieved when using only accessions with viability below 95%. While this might be counterintuitive, we show that the unintended variation in *p*_50_ estimates is approximately five times larger when using seeds with very high initial viabilities (>90%) compared to 75–85% (Figure 3). To date, comparative studies on seed longevity have restricted the initial viabilities from the bottom (>75%, >85%, or >90%) but have never set an upper limit (e.g., Probert et al., 2009; Merritt et al., 2014; Satyanti et al., 2018). While restricting the initial viabilities to a specific range reduces the unintended variation in *p*_50_ estimates, the estimates still contain some noise, just less than the *p*_50_ estimates currently in use. We thus consider this approach less optimal than the two solutions presented below.

### Solution 2: Use the rate of seed viability loss (σ)

The probit rate of seed viability loss (σ) is the direct measure of seed longevity in storage, is independent of the initial viability, and has occasionally been used as a measure of seed longevity (Hay and Probert, 1995; Kochanek et al., 2009; Carta et al., 2018). It represents the inverse of the slope of the probit generalized linear model used to describe the loss of seed viability in storage (Ellis and Roberts, 1980; Wolkis et al., 2025) and can be calculated using seeds with any reasonable initial viability. “Reasonable viability” in this context means a viability that enables reliable estimation of σ. If the initial viability is low and quickly drops to zero, there may be a lack of informative data points. Hay et al. (2022) already argued that σ is the optimal measure of seed longevity for comparative studies across species, accessions, and genotypes, but acknowledged that it is difficult to interpret without understanding the probit equation. Nevertheless, σ was reported alongside *p*_50_ in some previous studies, but comparisons among accessions were mostly based on *p*_50_ (Hay et al., 2010; Moravcová et al., 2022; but see Hay and Probert, 1995; Kochanek et al., 2009; Carta et al., 2018).

The probit, or “probability unit,” is rather abstract. In the context of seed aging, one would intuitively expect to translate σ to days in aging that are required for seeds to lose viability by a certain proportion. However, the rate of viability loss in percentage terms is not linear over time (Hay et al., 2019). Instead, the different viability intervals of, for example, 84.1% to 50%, 97.7% to 84.1%, and 99.9% to 99.7% all correspond to differences of one probit. In other words, a viability decline from 99.9% to 97.7%, 97.7% to 84.1%, or 84.1% to 50% takes the same number of days, described by σ (Figure 3). The loss of percentage viability is steepest in the middle of the curve, and the largest drop in percentage viability for one probit unit is from 69% to 31%, i.e., from +0.5 to −0.5 on the probit scale. In simple terms, σ can be described as the time needed for viability to decrease from, for example, 84% to 50% (Hay and Probert, 1995). The σ can be obtained directly from the seed viability equation as the inverse of the slope of the probit curve (specifically, −1/slope). The calculation for σ from the survival curve is included in the annotated R script provided in Appendix S2.

### Solution 3: Adjust *p*_50_ to a standardized initial viability

A third option is to use the rate of seed viability loss (σ) to calculate *p*_50_ standardized to a certain theoretical initial viability (e.g., 90%). This approach makes it possible to use any seed accession for which σ can be estimated, and subsequently calculate how many days it would take for viability (of this accession) to decrease from 90% to 50%. This calculation is very simple, and we provide an annotated R script in Appendix S2. This standardized *p*_50_ is proportional to σ, without any unintended noise, and could be more accessible as the concept is similar to the *p*_50_ estimates in current practice but avoids the unintended noise introduced by varying initial viabilities.

### Final remarks

Regardless of the parameter chosen to compare seed longevity in storage among accessions in a given study, it is essential to report all parameters of the seed viability curve—the theoretical initial viability (*K*_i_) and the probit rate of seed viability loss (σ). This will enable reciprocal recalculation among the estimates of *p*_50_, σ, and standardized *p*_50_ and allow comparisons in systematic reviews and meta‐analyses. An annotated R script is provided for these calculations in Appendix S2.

All parameters should be accompanied by confidence intervals. While reporting uncertainties was not the focus of this study, we emphasize that real experimental data are always affected by some noise and this causes uncertainty in the estimated parameters. Standard errors of the parameters estimated from the model are symmetrical around the mean only on probit scale as provided in the model output and become non‐symmetrical when back‐transformed to meaningful units. For example, a slope of −0.05 ± 0.01 SE corresponds to σ = 20 days, with a confidence interval of 16.67 to 25 days; it should be noted that this confidence interval is asymmetrical around the σ. Past studies on wild species mostly did not publish any uncertainties, with only a few of them reporting standard errors of the estimates, sometimes back‐transformed (e.g., Hay and Probert, 1995; Carta et al., 2018; Hay et al., 2019; White et al., 2023; Balasupramaniyam et al., 2025). In Appendix S2, we provide an annotated R code for the calculation of confidence intervals for all parameters.

Finally, we would like to encourage authors of future studies on seed longevity in storage to publish the raw germination data and experimental designs (Hay et al., 2022) and to share their data within open access initiatives (e.g., SeedArc; Fernández‐Pascual et al., 2023). This will ensure that their data are preserved and re‐used not only in the context of seed aging but also in explorations of the evolutionary and ecological drivers of seed germination.

## AUTHOR CONTRIBUTIONS

L.K., A.C., F.R.H., and A.B. conceived the study and reviewed the manuscript. A.B. and L.K. curated the data, conducted the formal analysis, and wrote the original draft. L.K. created the visualizations and acquired funding. A.B. supervised the project. All authors approved the final version of the manuscript.

## Supporting information


**Appendix S1.** Annotated R script for main analyses and figures.

**Appendix S2.** Annotated R script for additional calculations.

## Data Availability

All code and simulated data used for the analyses and figures in this study are provided in the Supporting Information and can be used to reproduce the results presented here.
