# Supplementary material for: Limitations of p 50 as a measure of seed longevity in comparative studies and the way forward
Source: Appl Plant Sci. 2025 Nov 29;13(6):e70032. doi: 10.1002/aps3.70032 (PMC12747544; doi:10.1002/aps3.70032)
Supplement: Supplementary file 1 — Appendix S1. Annotated R script for main analyses and figures. [file APS3-13-e70032-s002.pdf]

DOI 10.1002/aps3.70032

Appendix S1. Annotated R script for main analyses and figures.

Klepka, Lea (e-mail: lea.klepka@biologie.uni-marburg.de)
Bucharova, Anna (e-mail: anna.lampei-bucharova@uni-marburg.de)

2025-09-09

Content:

(0) Basic Code for Probit analysis (useful to understand further code)

(1) Relationship p50 ~ Ki (Figure 1A,B)

(2) Noise in p50 due to differences in initial viability (Figure 1B,D)

(3) Solutions to reduce/remove the noise in p50
  (3.1) Restrict range of initial viabilities (Figure 2)
  (3.2) Use sigma instead of p50 (Figure 3)
  (3.3) Standardized p50 (as illustrated in Figure 2A)

Load packages

```
library(drc)
library(MASS)
library(dplyr)
library(ggplot2)
library(cowplot)
library(arm)
```

Set consistent sizes for plots

```
point.size = 0.8
line.size = 0.75
lab.size = 12
text.size = 8
```

DOI 10.1002/aps3.70031

(0) BASIC CODE FOR VISUALIZING SEED LONGEVITY MODEL RESULTS

0.1 Probit transformation and backtransformation

```
qnorm(0.999) # function to transform percentage probability to probit scale
```

```
[1] 3.090232
```

```
pnorm(-2) # function tor back-transformation from probit to percentage probability
```

```
[1] 0.02275013
```

```
# (pnorm(0)= 0.5)) 50% probability coresponds to a probit-value of 0
```

Note: A viability of 100% corresponds to "Inf" on the probit scale and must be avoided in computations.

0.2 Relationship: Percentage - Probit

```
seed_viability <- seq(from=0.001, to=0.999, by=0.001) # generate vector with
                                                       # seed viability between 0.001 and 0.999

# generate dataframe with seed viability and corresponding probit value
Transformation <- data.frame(
  seed_viability = seed_viability,
  probit = qnorm(seed_viability)) # see qnorm() function above

# plot relationship between seed viability (%) and corresponding probit value
p_transformation <- ggplot(data = Transformation, aes(x = seed_viability, y = probit)) +
  geom_line(size = line.size, colour = "#FF7F50") +
  labs(x = "Seed viability", y = "Probit") +
  scale_x_continuous(labels = scales::percent) +
  scale_y_continuous(breaks = seq(-3,3,1)) +
  theme_classic()

p_transformation # show plot
```

DOI 10.1002/aps3.70031

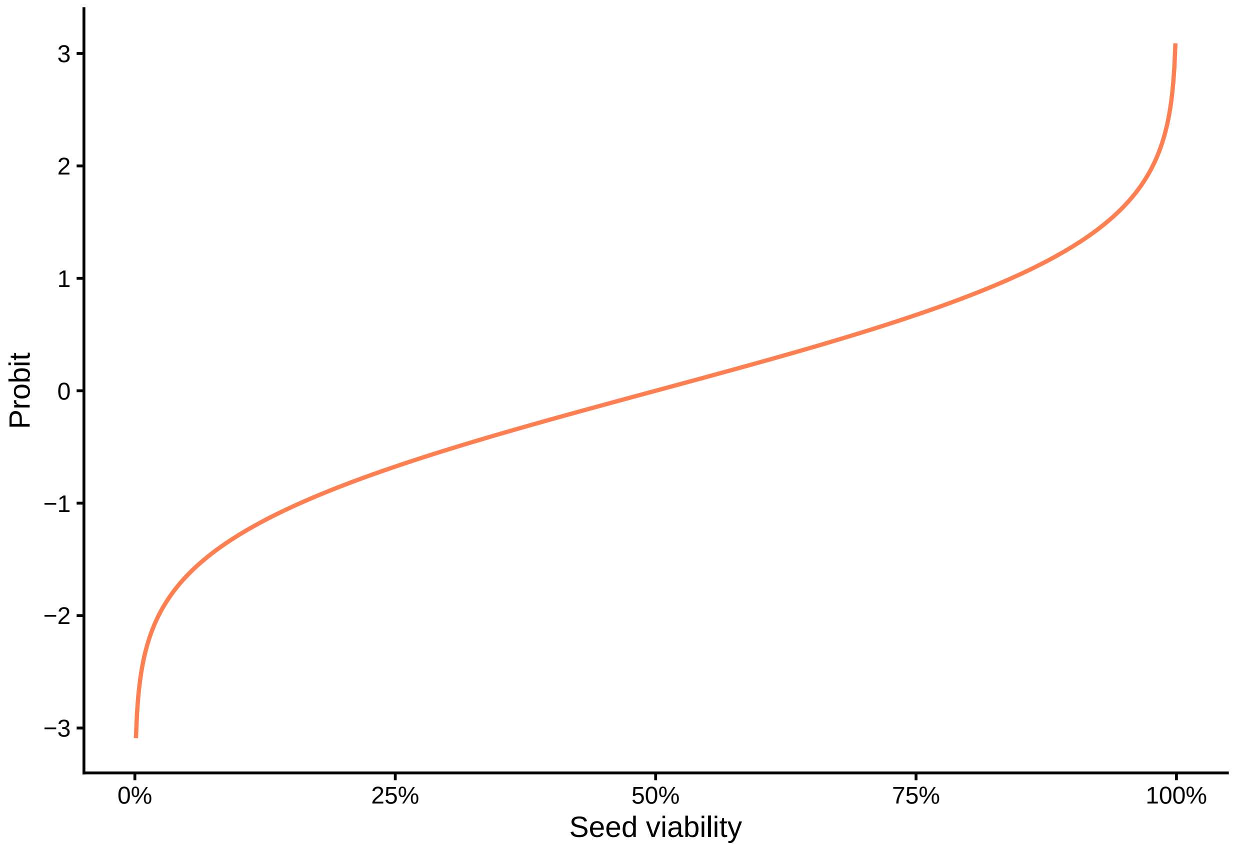

0.3 Illustrate seed viability in storage in percentage and probits

Draw a curve that illustrates seed viability (%) in storage

Sigma = time in storage needed to reduce viability by 1 probit

```
aging_in_sigma <- seq(from=3, to=-3, by=-0.01) # Percentage of germination success
                                                # range from 0.1349898% to 99.86501%
                                                # corresponds to probit range -3 to 3
time_in_sigma <- (-1*aging_in_sigma)+3 # shift range from -3 to 3 (contains negative values)
                                        # to 0 to 6 (contains only positive values)
seed_viability <- pnorm(aging_in_sigma) # calculate seed viability (back transformation from probit)
Ki <- qnorm(seed_viability) # probit transformation (from percentage to probit)

# generate dataframe to illustrate seed survival
Seed_survival <- data.frame(
  time_in_sigma = time_in_sigma,
  seed_viability = seed_viability)

# Plot Seed survival curve (relationship between seed viability (%) and time in aging (sigma))
p_seed_survival <- ggplot(data = Seed_survival, aes(x = time_in_sigma, y = seed_viability)) +
  geom_line(size = line.size, colour = "#FF7F50") +
  labs(x = "Time in aging (sigma)", y = expression("Seed viability (%)")) +
  scale_y_continuous(labels = scales::percent) +
  scale_x_continuous(breaks = seq(min(Seed_survival$time_in_sigma),
```

DOI 10.1002/aps3.70031

```
                                max(Seed_survival$time_in_sigma), 1)) +
  theme_classic() +
  theme(
    axis.title.x = element_text(size = lab.size),
    axis.title.y = element_text(size = lab.size),
    axis.text.x = element_text(size = text.size),
    axis.text.y = element_text(size = text.size),
    plot.margin = margin(c(0,0,0,0))
  )

p_seed_survival # show plot
```

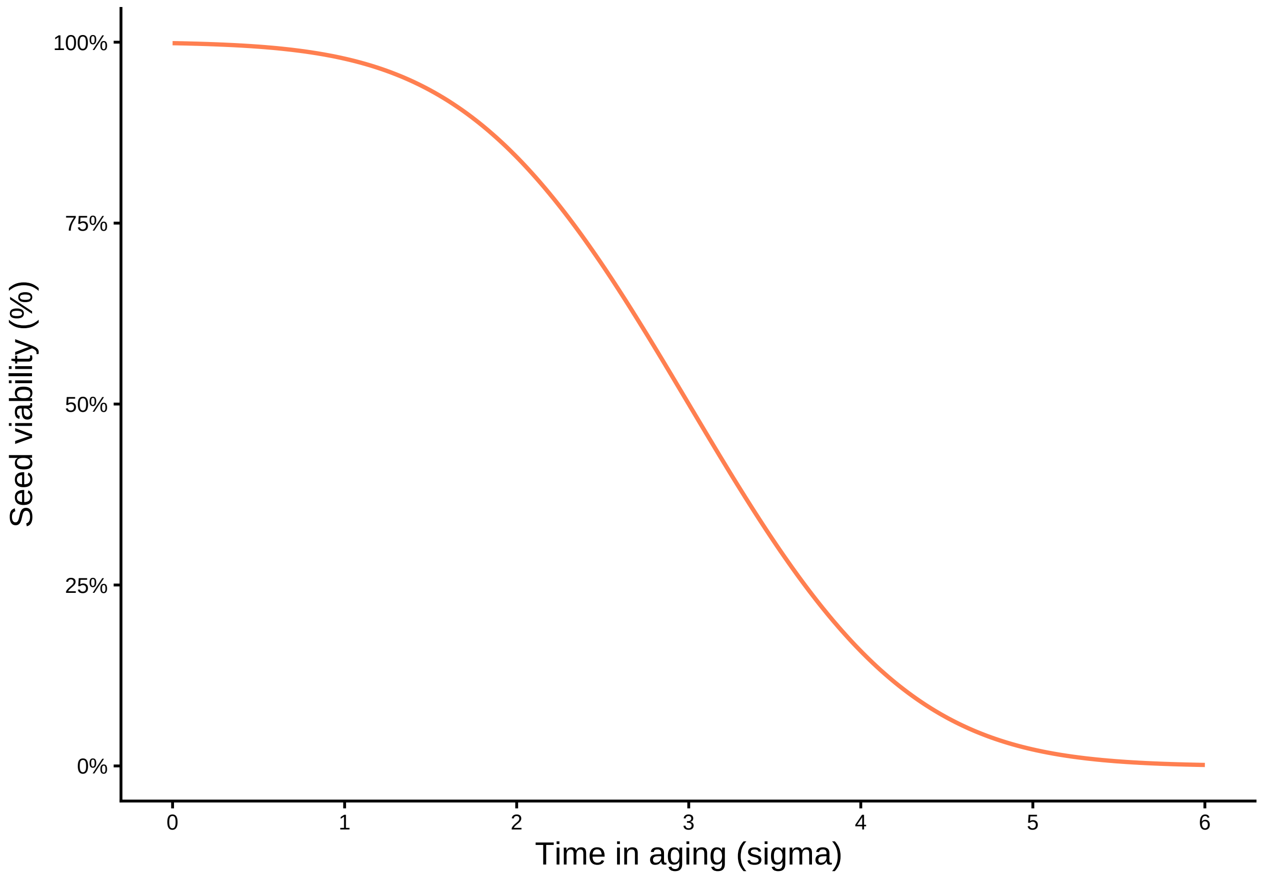

Draw a curve that illustrates seed viability (probits) in storage

Relationship between seed viability (probit) and time in aging (sigma))

```
p_seed_survival_probit <- ggplot(data = Seed_survival,
                               aes(x = time_in_sigma, y = qnorm(seed_viability))) +
  geom_line(size = line.size, colour = "#FF7F50") +
  labs(x = "Time in aging (sigma)", y = "Seed viability (probit)") +
  scale_x_continuous(breaks = seq(min(Seed_survival$time_in_sigma),
                                max(Seed_survival$time_in_sigma), 1)) +
  theme_classic() +
  theme(
```

DOI 10.1002/aps3.70031

```
    axis.title.x = element_text(size = lab.size),
    axis.title.y = element_text(size = lab.size),
    axis.text.x = element_text(size = text.size),
    axis.text.y = element_text(size = text.size),
    plot.margin = margin(c(0,0,0,0))
  )

p_seed_survival_probit # show plot
```

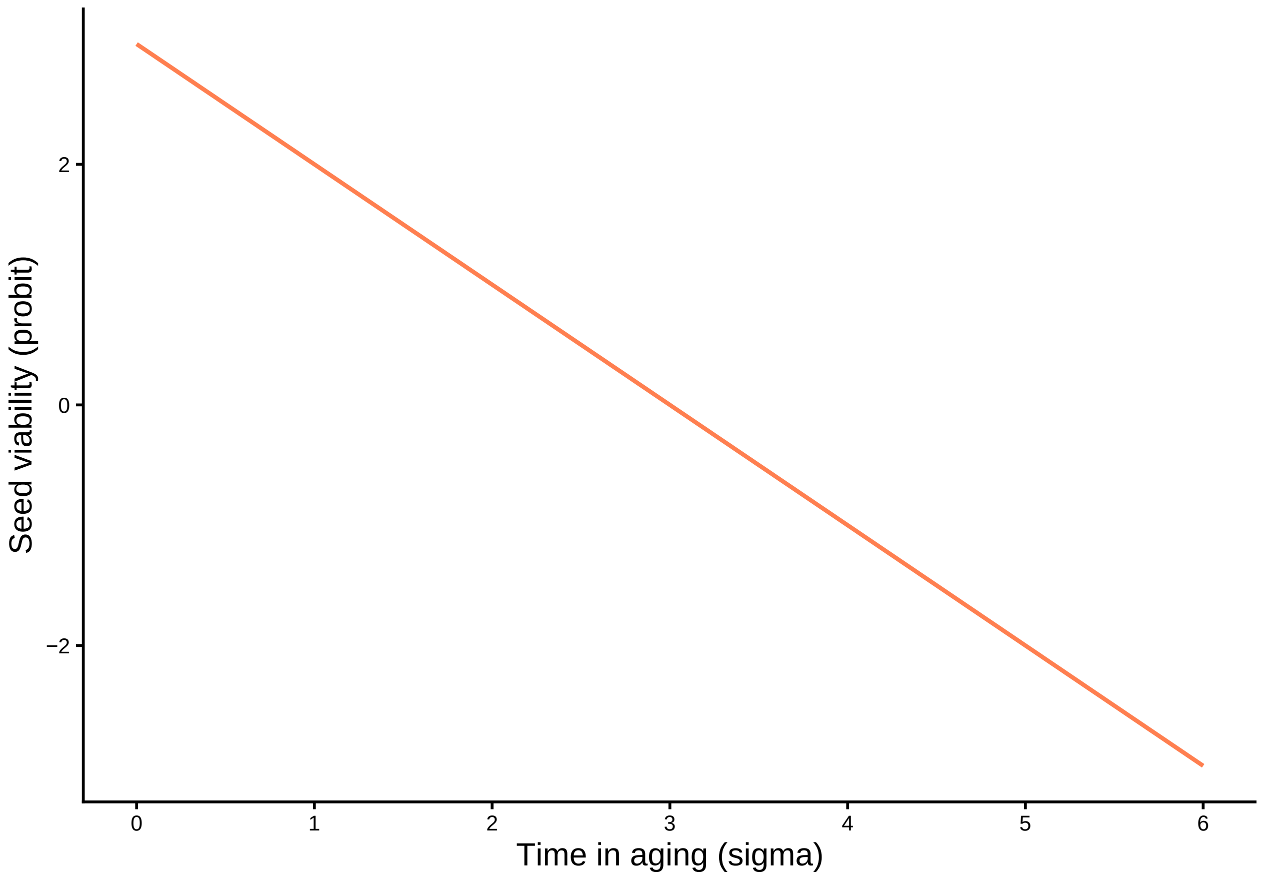

Combine seed survival plots to compare viability in percentage and probit

```
p_0 <- plot_grid(p_seed_survival, p_seed_survival_probit, ncol = 2,
                 labels = c("A", "B"), label_size = 12, label_x = 0, label_y = 1,
                 rel_widths = c(0.5, 0.5))

p_0 # show plot
```

DOI 10.1002/aps3.70031

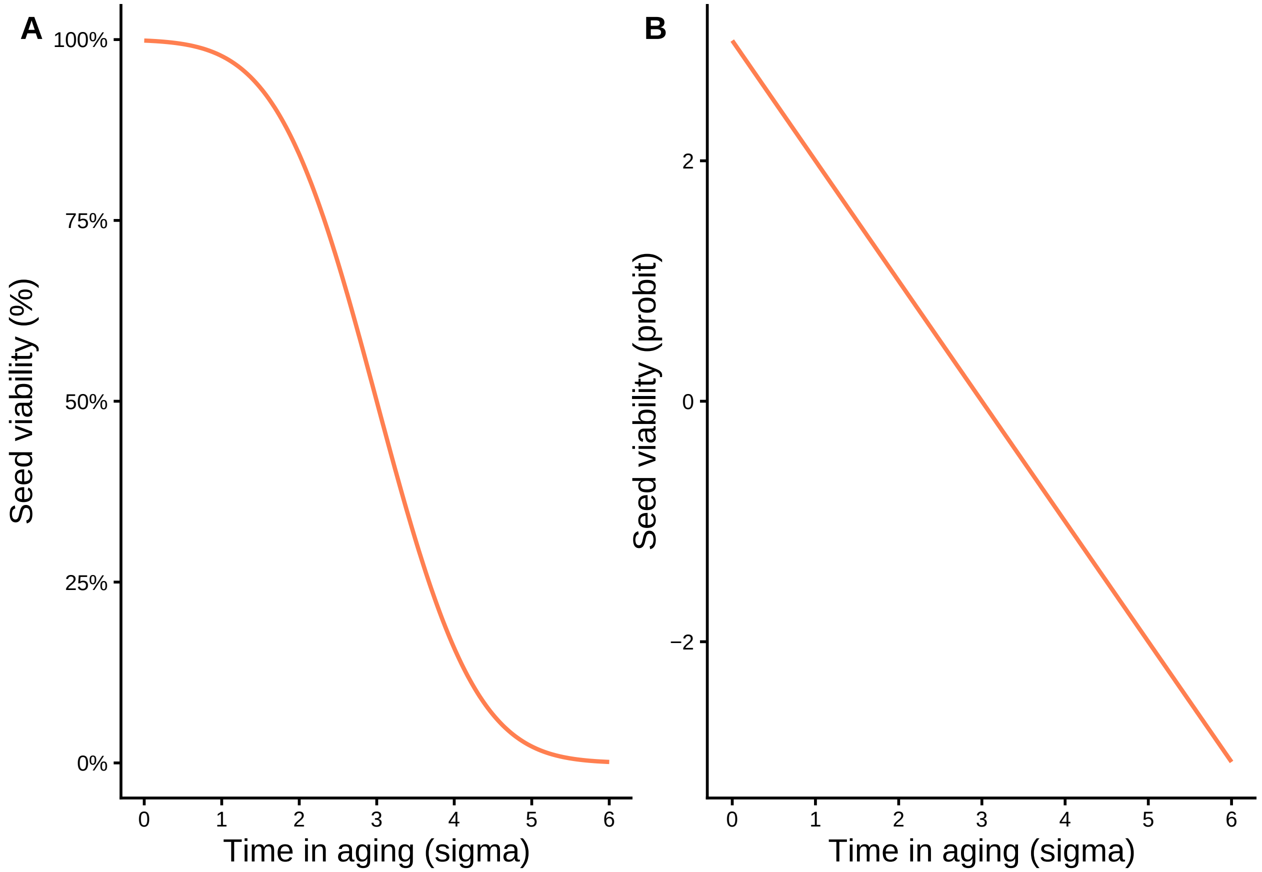

(1) RELATIONSHIP BETWEEN P50 AND INITIAL VIABILITY (Ki)

1.1 Simulate hypothetical species

```
sigma_days <- 20 # set fixed sigma in days
sigma_probit = -1/sigma_days # slope in probit units per day
                             # (after 1 day, probit decreases by 1/20)

viabilities <- seq(from=0.85, to=0.999, by=0.001) # sequence of initial viabilities (in percentage)
                                                   # from 0.85 to 0.999
Ki.probit <- qnorm(viabilities) # transformation of viabilities from percentage to probit
p50 <- Ki.probit*sigma_days # calculate P50 applying equation (2): p50 = Ki*sigma

# create dataframe for p50~Ki relationship
Relationship_p50_Ki <- data.frame(
  viability = viabilities,
  p50 = p50)
```

DOI 10.1002/aps3.70031

1.2 Calculate minimum and maximum p50 and ratio

```
p50_min <- round(min(p50),2)
p50_max <- round(max(p50),2)
p50_ratio <- p50_max/p50_min

# show results
p50_min
```

```
[1] 20.73
```

```
p50_max
```

```
[1] 61.8
```

```
p50_ratio
```

```
[1] 2.981187
```

1.3 Figure 1 B: Relationship between p50 and the initial seed viability

```
p_1B <- ggplot(data = Relationship_p50_Ki, aes(x = viability, y = p50)) +
  geom_line(colour = "#FF7F50", size=line.size) +
  labs(x= expression("Initial viability - "~ K[i]~" (%)"),
       y = expression(p[50] ~ " (days)")) +
  scale_x_continuous(labels = scales::percent, limits = c(0.85,1.01)) +
  scale_y_continuous(limits = c(0,70)) +
  theme_classic() +
  theme(
    axis.title.x = element_text(size = lab.size),
    axis.title.y = element_text(size = lab.size),
    axis.text.x = element_text(size = text.size),
    axis.text.y = element_text(size = text.size),
    plot.margin = margin(c(0,0,0,0))
  )

p_1B # show plot
```

DOI 10.1002/aps3.70031

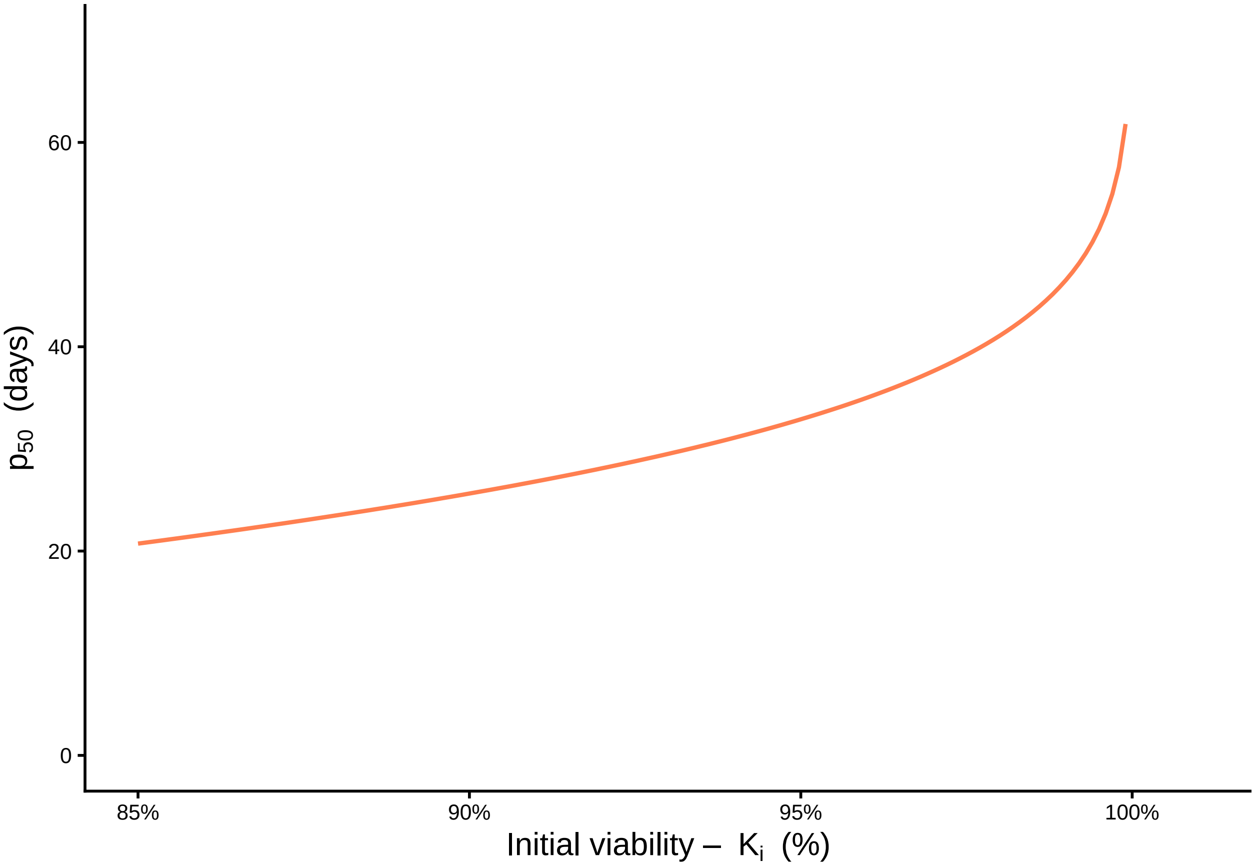

1.4 Figure 1A: Seed viability loss

with sigma = 20 and 4 different initial viabilities. This code is rather long because it contains many design elements.

```
sigma_days <- 20 # set fixed sigma in days
sigma_probit = -1/sigma_days # slope in probit units per day (after 1 day, probit decreases by 1/20)

# generate dataframe for hypothetical seed aging experiment
days_in_aging <- seq(0,100,1) # sequence of aging duration (between 0 and 100 days)
viability_probit <- qnorm(0.9991) + sigma_probit*days_in_aging # calculate seed viability for each
                                                                # aging duration in probit;
                                                                # Sigma_probit = viability loss per day
viability <- pnorm(viability_probit) # backtransformation to percentage

One_spec <- data.frame(
  days_in_aging = days_in_aging,
  viability = viability)

# generate an example p50 for 4 defined initial viabilities (Ki)
Ki_percentage <- c(0.85,0.90,0.95,0.999) # vector of initial viabilities (in percentage)
Ki_probit <- qnorm(Ki_percentage) # transformation of viabilities from percentage to probit
p50 <- Ki_probit*sigma_days # calculate P50 applying equation (2):
# p50 = Ki*sigma (one p50 value for each initial viability)
```

DOI 10.1002/aps3.70031

```r
curve_start <- 0.9991 # theoretical maximum viability
# --> this is where the seed survival curve starts (the same for all initial viabilities)
# calculate the number of days it takes to decay from theoretical maximum viability (curve_start)
# to given initial viability - for plotting
x_viabs <- (qnorm(curve_start)-qnorm(Ki_percentage))*sigma_days # seed viability equation applied:
                                                                # p = (Ki-viability)*sigma

# define ends of grey lines for plotting
y_ends <- c(0.5, 0.43, 0.36, 0.29)

# labels - how long it takes to decay to p50 from given initial viability
p50lab <- paste0("p[50] == ", round(qnorm(Ki_percentage)*sigma_days,1), " * ' days'")
# calculation of p50 for given initial viability
viabslab <- paste0("K[i]~'(%)' == ", Ki_percentage*100, " * '%'")

# plot relationship between seed viability an aging duration
p_1A <- ggplot(data = One_spec, aes(x=days_in_aging, y=viability)) +
  geom_point(shape = 16, y = 0.5, x = qnorm(curve_start)*sigma_days,
             col="#E66A3A", size = 4) + # point to 50% germination (p50 = Ki*Sigma)
  geom_segment(aes(x=qnorm(curve_start) * sigma_days, xend=qnorm(curve_start) * sigma_days,
                   y=0.5,yend=min(y_ends)), color="grey80") + # vertical line at p50
  geom_line(size=line.size) # seed survival curve

# add labels and lines for each of the example initial viabilities
for (i in 1:length(Ki_percentage)) {
  segment_data <- data.frame(x = x_viabs[i], # how long it takes to reach given initial viability
                             y = y_ends[i], # lower ends of vertical lines
                             yend = Ki_percentage[i]-0.002, # upper end of vertical lines
                             p50 = qnorm(curve_start) * sigma_days, # p50 = Ki* sigma
                             labs = p50lab[i], # labels
                                               # (how long it takes to decay to p50 from
                                               # given initial viability)
                             xtext = 51.5, # Position of text
                             ytext = y_ends[i] + 0.02, # Position of text (0.03 above arrow line)
                             labsK = viabslab[i], # label for Ki(%)
                             xK = x_viabs[i], # Position of given initial viability
                             yK = Ki_percentage[i] + 0.02 ) # Position of given initial viability
                                                            # (at y=initial viability -0.08 down)
  p_1A <- p_1A +
    geom_segment(data = segment_data, aes(x = x, xend = x,
                                          y = y, yend = yend),
                 color = "grey80", size = line.size) + # vertical lines for
                                                       # each given initial viability
    geom_segment(data = segment_data, aes(x = x, xend = p50,
                                          y = y, yend = y),
                 color="#828282", size = 0.6,
                 arrow = arrow(ends = "both",length=unit(0.02,"npc"))) + # horizontal lines + arrow
    geom_text (data = segment_data, aes(x = xtext, y = ytext, label = labs),
               parse = T, size = text.size, size.unit = "pt") + # text: p50 = 20.7 days, ...
    geom_text (data = segment_data, aes(x = xK, y = yK, label=labsK),
               hjust = 0, parse = T, size = text.size, size.unit = "pt") # text: given initial
                                                                         # viability
}
```

DOI 10.1002/aps3.70031

```
p_1A <- p_1A +
  labs(x = "Days in aging", y = expression("Initial viability - "~ K[i]~" (%)")) +
  scale_y_continuous (labels = scales::percent) +
  theme_classic() +
  theme(
    axis.title.x = element_text(size = lab.size),
    axis.title.y = element_text(size = lab.size),
    axis.text.x = element_text(size = text.size),
    axis.text.y = element_text(size = text.size),
    plot.margin = margin(c(0,0,0,0))
  )

p_1A # show plot
```

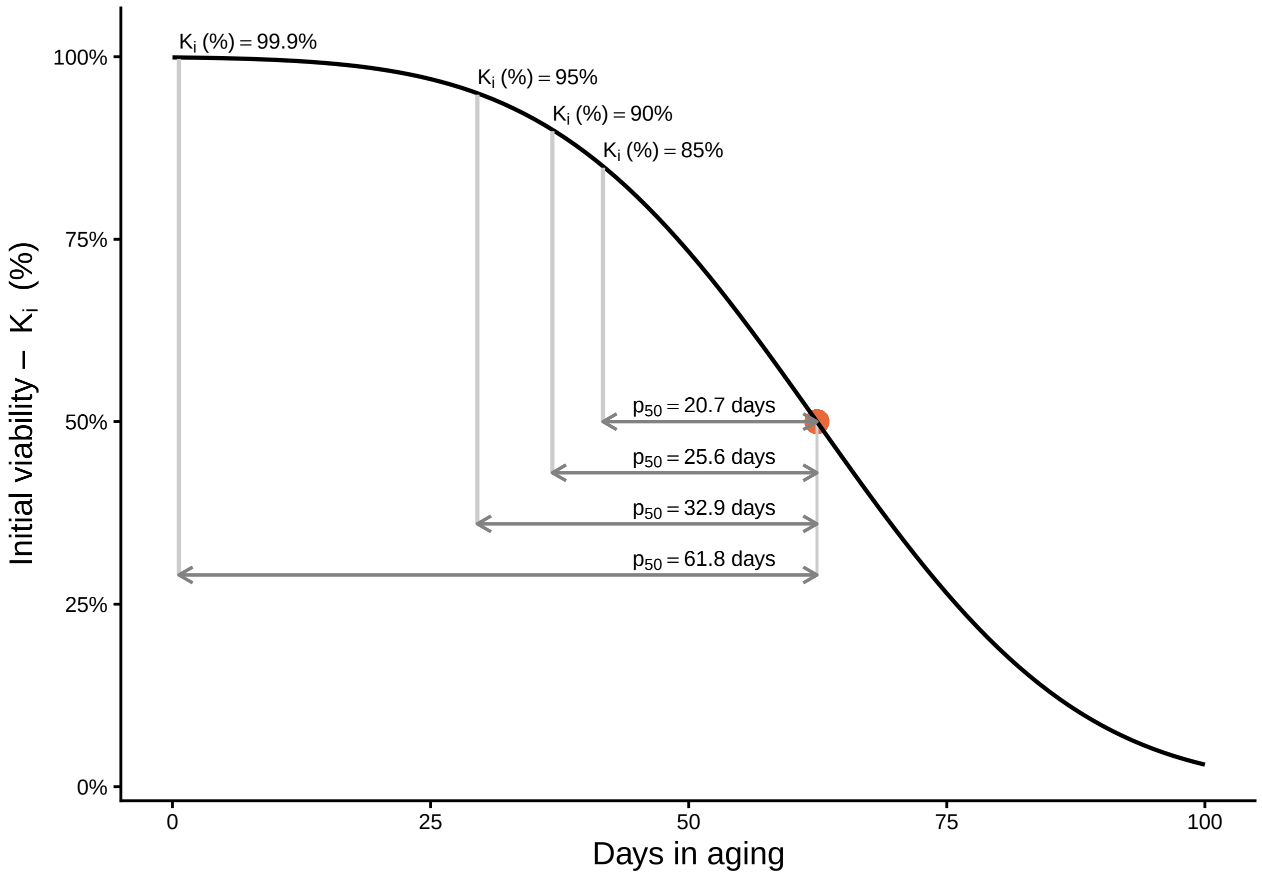

(2) NOISE IN P50 DUE TO DIFFERENCES IN INITIAL VIABILITY

2.1 Simulate 500 seed lots with random initial viabilities and random sigma-values

DOI 10.1002/aps3.70031

```r
lot_number <- 500 # number of random seed lots
viability <- runif(lot_number, min = 0.85, max = 0.999) # random initial viabilities for
                                                          # each seed lot varying between
                                                          # min (0.85) and max (0.999)
sigma <- runif(lot_number, min = 1, max = 50) # random values of sigma (in days) for
                                              # each seed lot varying between
                                              # min (1 day) and max (50 days)

# create dataframe for simulated 500 random seed lots with generated initial viabilities and sigma
Simdat <- data.frame(
  viability = viability,
  sigma = sigma)
Simdat$ID <- row(Simdat)[,1] # assign IDs to simulated species

# calculate p50 for simulated seed lots with original initial viability and fixed Ki
Simdat <- Simdat %>%
  mutate(
    p50 = qnorm(viability) * sigma,
    p50_0.75 = qnorm(0.75) * sigma,  # Fixed Ki(%) at 0.75
    p50_0.80 = qnorm(0.80) * sigma,  # Fixed Ki(%) at 0.80
    p50_0.85 = qnorm(0.85) * sigma,  # Fixed Ki(%) at 0.85
    p50_0.90 = qnorm(0.90) * sigma,  # Fixed Ki(%) at 0.90
    p50_0.95 = qnorm(0.95) * sigma,  # Fixed Ki(%) at 0.95
    p50_0.999 = qnorm(0.999) * sigma # Fixed Ki(%) at 0.999
  )
```

Colour gradients for plots

```r
# function for plots
create_gradient <- function(low, high) {
  scale_color_gradient2(
    low = "#3B4994", mid = "#FF7F50", high = "#FFC300",
    midpoint = 0.93, limits = c(low, high)
  )
}

# function for legend in Figure 1
create_gradient_label <- function(low, high) {
  scale_fill_gradient2(
    low = "#3B4994", mid = "#FF7F50", high = "#FFC300",
    midpoint = 0.93, limits = c(low, high)
  )
}

# create dummy dataframe for gradient legend (Figure 1)
Dummy_data <- data.frame(
  x = rep(seq(min(Simdat$viability), max(Simdat$viability),
            length.out = 100), each = 100),  # Create a grid for x
  y = rep(seq(min(Simdat$viability), max(Simdat$viability),
            length.out = 100), times = 100)  # Create a grid for y
)
```

DOI 10.1002/aps3.70031

```
# Create legend (Figure 1)
p_legend_gradient <- ggplot(Dummy_data, aes(x = x, y = y, fill = y)) +
  geom_tile() +  # create a grid of rectangles
  create_gradient_label(min(Simdat$viability), max(Simdat$viability)) + # create gradient
  ylim(min(Simdat$viability), 1.2) +
  scale_y_continuous(labels = scales::percent, name = NULL, position = "right") + # Remove y-axis label
  scale_x_continuous(
    name = expression("Initial viability - " ~ K[i] ~ "(%)") ,breaks = NULL, # Remove x-axis labels
                                                                              # and ticks
    position = "top") +
  theme_classic() +
  theme(
    axis.text.y = element_text(size = text.size),  # Hide axis text
    axis.text.x = element_blank(),  # Hide axis text
    axis.title.x = element_text(size = text.size, hjust = 0),
    axis.ticks = element_line(),  # Keep the ticks but remove the axis line
    axis.ticks.y = element_line(),  # Keep y-axis ticks
    axis.line = element_blank(),  # Remove the axis line
    panel.border = element_blank(),  # Remove the panel border
    legend.position = "none",
    plot.margin = margin(80,5,80,10),
  )

p_legend_gradient # show legend
```

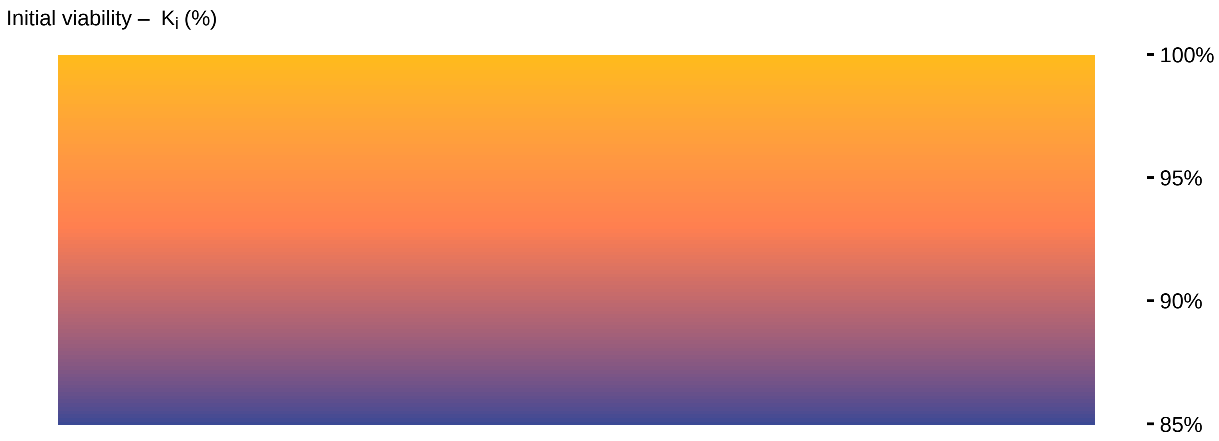

DOI 10.1002/aps3.70031

2.2 Figure 1C: Noise in p50

using seed lots with initial viabilities varying between 85 and 99.9%

```
p_1C <- ggplot(data = Simdat, aes(x = sigma, y = p50, color = viability)) +
  geom_line(aes(x = sigma, y = p50_0.85), color = "#828282", size = line.size) +
  geom_line(aes(x = sigma, y = p50_0.999), color = "#828282", size = line.size) +
  geom_point(shape = 19, alpha = 0.6, size = point.size) +
  create_gradient(min(Simdat$viability), max(Simdat$viability)) +  # Use whole gradient
  ylim(0, 150) +
  labs(x = "Sigma", y = expression(p[50] * " (days)")) +
  theme_classic() +
  theme(
    axis.title.x = element_text(size = lab.size),
    axis.title.y = element_text(size = lab.size),
    axis.text.x = element_text(size = text.size),
    axis.text.y = element_text(size = text.size),
    plot.margin = margin(c(0,0,0,0))
  )

p_1C # show plot
```

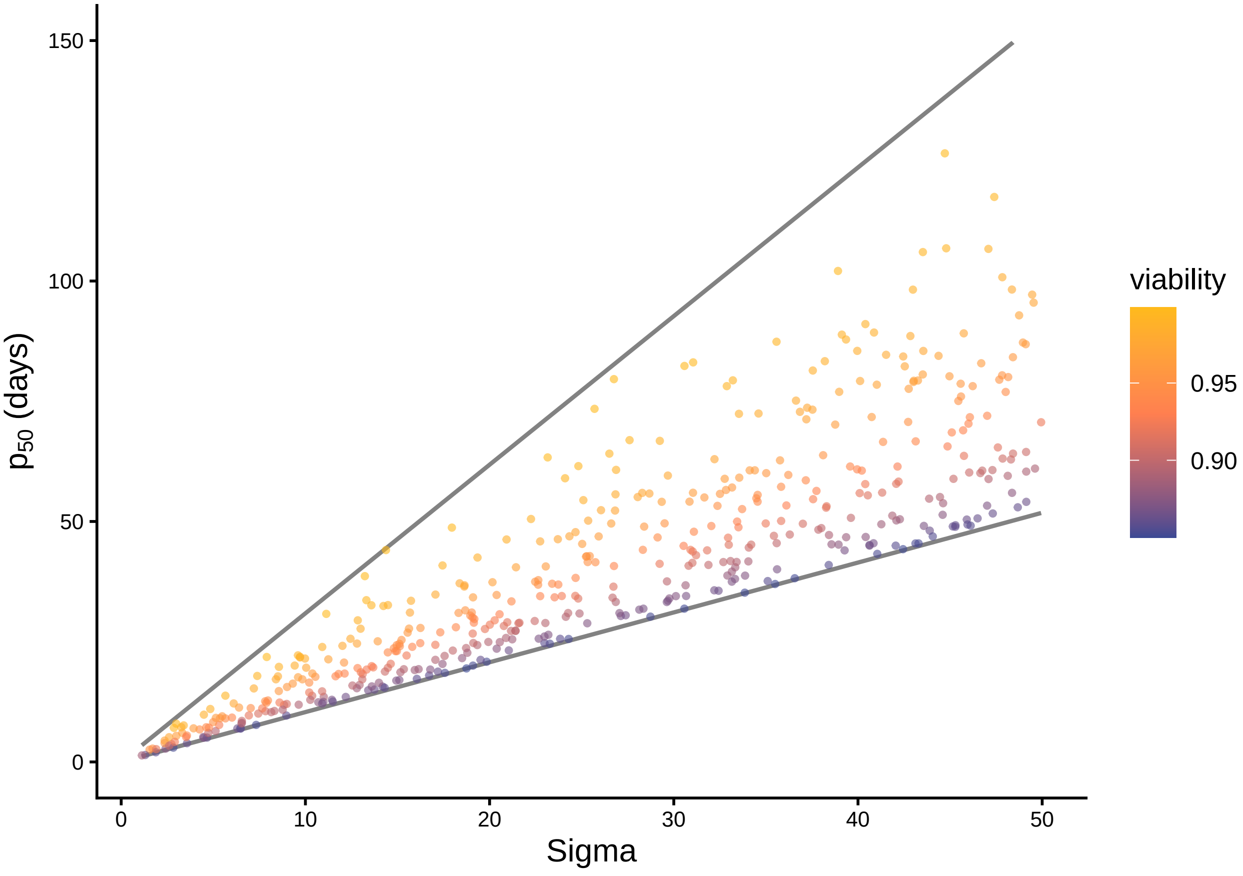

2.3 Figure 1D: Log-transformation of p50

Same as Figure 1C, but with log-transformed y-axis

DOI 10.1002/aps3.70031

```
p_1D <- ggplot(data = Simdat, aes(x = sigma, y = p50, color = viability)) +
  geom_line(aes(x = sigma, y = p50_0.85), color = "#828282", size = line.size) +
  geom_line(aes(x = sigma, y = p50_0.999), color = "#828282", size = line.size) +
  geom_point(shape = 19, alpha = 0.6, size = point.size) +
  create_gradient(min(Simdat$viability), max(Simdat$viability)) +  # Use whole gradient
  ylim(0, 150) +
  scale_y_log10(breaks = c(0, 0.1, 10, 100, 150)) +
  labs(x = "Sigma", y = expression(log(p[50]) * " (days)")) +
  theme_classic() +
  theme(
    axis.title.x = element_text(size = lab.size),
    axis.title.y = element_text(size = lab.size),
    axis.text.x = element_text(size = text.size),
    axis.text.y = element_text(size = text.size),
    plot.margin = margin(c(0,0,0,0))
  )

p_1D # show plot
```

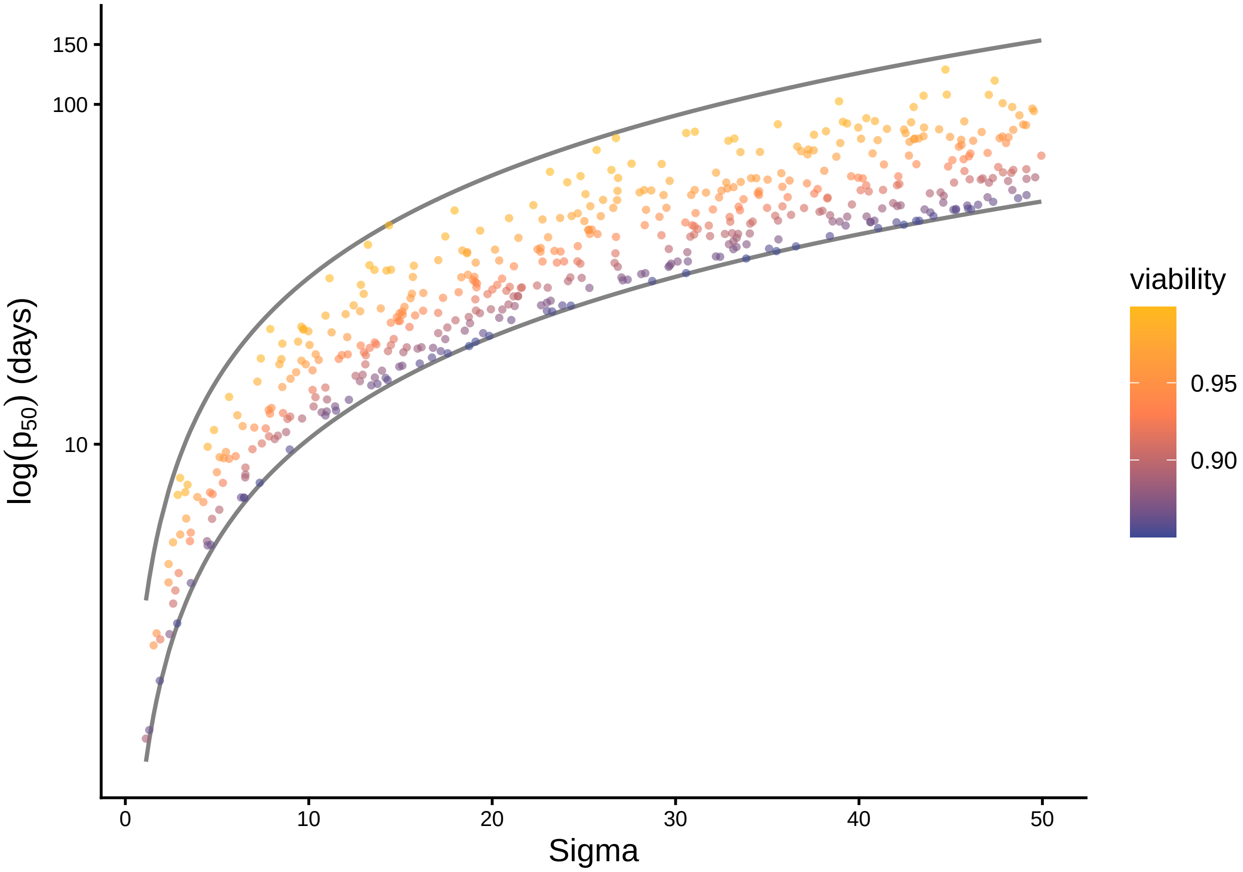


```
# the relationship between p50 (if Ki(%)=0.85) and p50 (if Ki(%)=0.999)
unique(Simdat$p50_0.999/Simdat$p50_0.85)
```

```
[1] 2.981602 2.981602 2.981602
```

DOI 10.1002/aps3.70031

```
# the relationship between p50 (if Ki=0.85) and p50 (if Ki=0.999), on log-scale
unique(log(Simdat$p50_0.999)-log(Simdat$p50_0.85))
```

```
[1] 1.092461 1.092461 1.092461 1.092461 1.092461
```

2.4 Combine Figure 1

```
# Remove legend from p_2C and p_2D
p_1C_nolegend <- p_1C + theme(legend.position = "none")
p_1D_nolegend <- p_1D + theme(legend.position = "none")

p_1 <- plot_grid(
  plot_grid(p_1A, p_1B, ncol = 2,
            labels = c("A", "B"), label_size = 12, label_x = 0, label_y = 1,
            rel_widths = c(0.7, 0.3)),  # Adjust the width ratio for the first row
  plot_grid(p_1C_nolegend, p_1D_nolegend, p_legend_gradient, ncol = 3,
            labels = c("C", "D", ""), label_size = 12, label_x = 0, label_y = 1.01,
            rel_widths = c(0.42, 0.42, 0.16)),
  nrow = 2
) + theme(plot.margin = margin(0,15,0,0))

p_1 # show plot
```

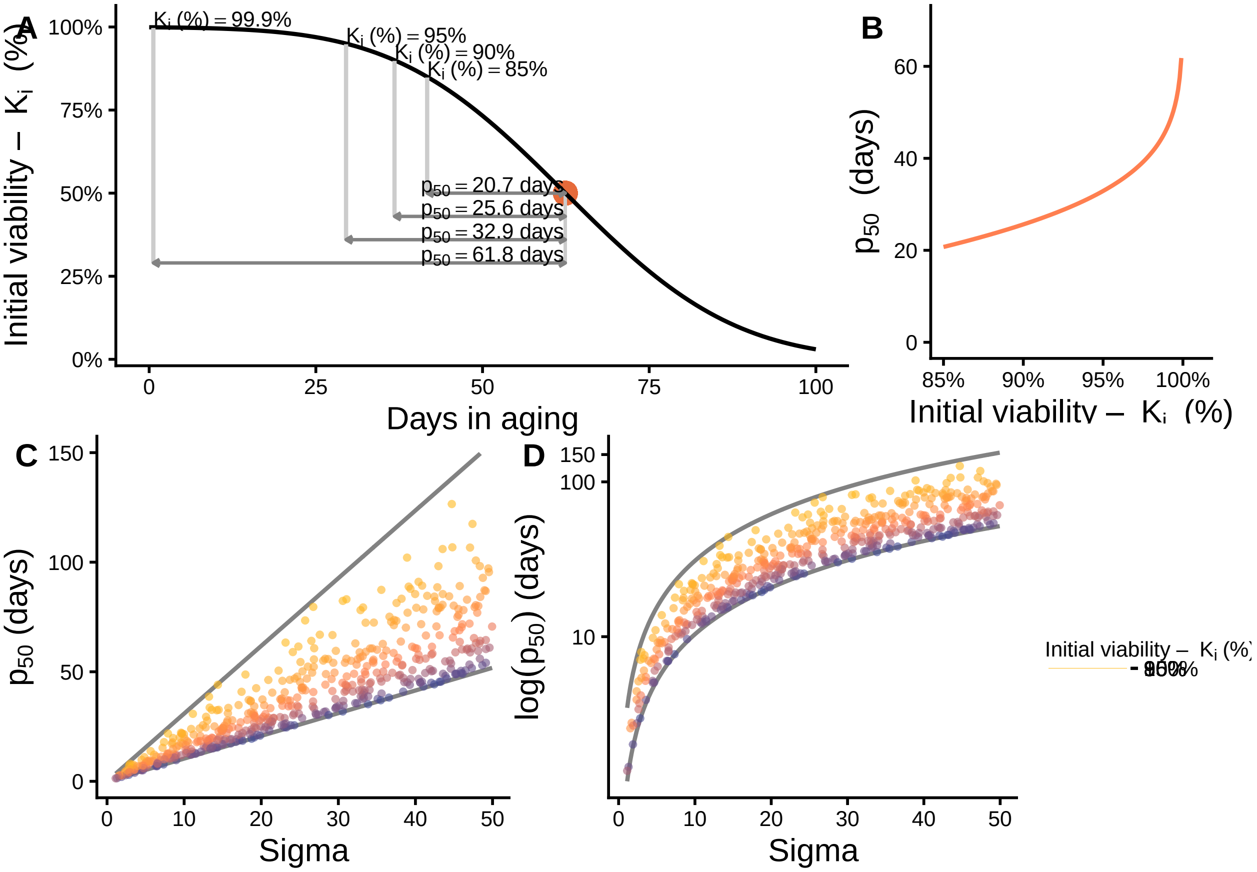

DOI 10.1002/aps3.70031

```
# save plot (the saved plots look identical to the figures in the paper)
# ggsave(file = "your/path/Figure1.pdf", plot = p_1, width = 184, height = 200, units = "mm", dpi=600)
```

(3) SOLUTIONS TO REDUCE/REMOVE THE NOISE IN P50

3.1 Figure 2: Restrict range of initial viabilities

```
# simulate 500 seed lots with random initial viabilities and random sigma-values (same as in (2))
lot_number <- 500 # number of random seed lots
sigma <- runif(lot_number, min = 1, max = 50) # random values of sigma (in days) for each seed lot
                                              # varying between min (1 day) and max (50 days)

# create dataframe for simulated 500 random seed lots with generated initial viabilities and sigma
Simdat <- data.frame(sigma = sigma)
Simdat$ID <- row(Simdat)[,1] # assign IDs to simulated species

# calculate p50 for simulated seed lots that they would have had if Ki(%) was fixed
Simdat <- Simdat %>%
  mutate(
    p50_0.75 = qnorm(0.75) * sigma,  # Fixed Ki(%) at 0.75
    p50_0.80 = qnorm(0.80) * sigma,  # Fixed Ki(%) at 0.80
    p50_0.85 = qnorm(0.85) * sigma,  # Fixed Ki(%) at 0.85
    p50_0.90 = qnorm(0.90) * sigma,  # Fixed Ki(%) at 0.90
    p50_0.95 = qnorm(0.95) * sigma,  # Fixed Ki(%) at 0.95
    p50_0.999 = qnorm(0.999) * sigma # Fixed Ki(%) at 0.999
  )
```

Illustrate the relationship between sigma and p50 if initial viability is fixed

```
p_2A.1 <- ggplot(data = Simdat, aes(x = sigma, y = p50)) +
  geom_line(aes(x = sigma, y = p50_0.75), color = "#828282", size = line.size) + # for Ki(%) = 0.75
  geom_line(aes(x = sigma, y = p50_0.80), color = "#828282", size = line.size) + # for Ki(%) = 0.80
  geom_line(aes(x = sigma, y = p50_0.85), color = "#828282", size = line.size) + # for Ki(%) = 0.85
  geom_line(aes(x = sigma, y = p50_0.90), color = "#828282", size = line.size) + # for Ki(%) = 0.90
  geom_line(aes(x = sigma, y = p50_0.95), color = "#828282", size = line.size) + # for Ki(%) = 0.95
  geom_line(aes(x = sigma, y = p50_0.999), color = "#828282", size = line.size) + # for Ki(%) = 0.999

 annotate("text", x = 52, hjust = 0, y = max(Simdat$p50_0.75),
          label = "K[i]~'(%)'==75*'%'", parse = TRUE,
          size = text.size, size.unit = "pt") +
  annotate("text", x = 52, hjust = 0, y = max(Simdat$p50_0.80),
          label = "K[i]~'(%)'==80*'%'", parse = TRUE,
          size = text.size, size.unit = "pt") +
  annotate("text", x = 52, hjust = 0, y = max(Simdat$p50_0.85),
          label = "K[i]~'(%)'==85*'%'", parse = TRUE,
          size = text.size, size.unit = "pt") +
  annotate("text", x = 52, hjust = 0, y = max(Simdat$p50_0.90),
          label = "K[i]~'(%)'==90*'%'", parse = TRUE,
```

DOI 10.1002/aps3.70031

```
          size = text.size, size.unit = "pt") +
  annotate("text", x = 52, hjust = 0, y = max(Simdat$p50_0.95),
          label = "K[i]~'(%)'==95*'%'", parse = TRUE,
          size = text.size, size.unit = "pt") +
  annotate("text", x = 52, hjust = 0, y = max(Simdat$p50_0.999),
          label = "K[i]~'(%)'==99.9*'%'", parse = TRUE,
          size = text.size, size.unit = "pt") +
  xlim(0, 65) +
  labs(x = "Sigma", y = expression(p[50] * " (days)")) +
  theme_classic() +
  theme(
    axis.title.x = element_text(size = lab.size),
    axis.title.y = element_text(size = lab.size),
    axis.text.x = element_text(size = text.size),
    axis.text.y = element_text(size = text.size),
    plot.margin = margin(c(0,0,0,0))
  )

p_2A.1 # show plot
```

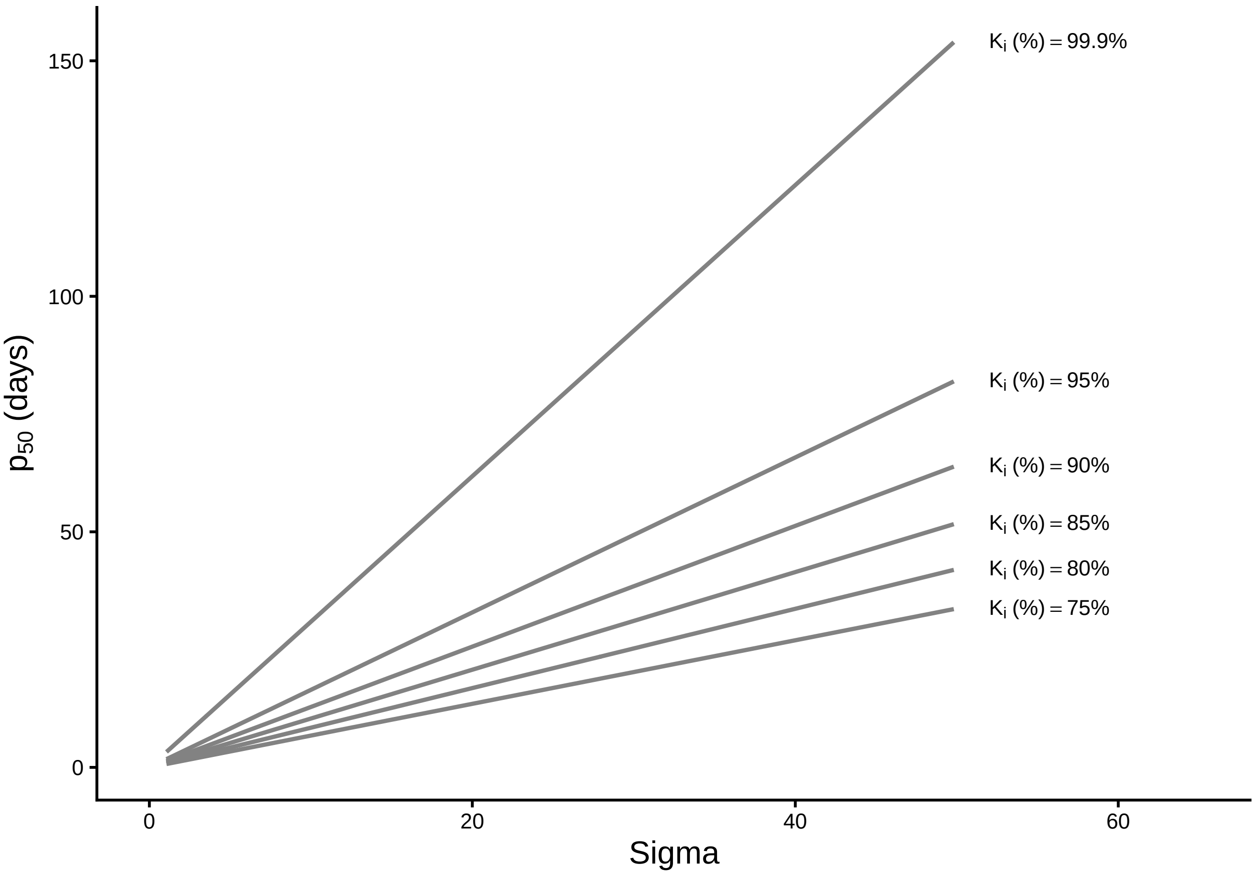

DOI 10.1002/aps3.70031

Illustrate the relationship between sigma and p50 if initial viability is limited to intervals

```
# interval Ki(%) 0.75-0.85
Ki_0.85_0.75 <- round(((Simdat$p50_0.85-Simdat$p50_0.75)/Simdat$sigma)[1], 2)
# interval Ki(%) 0.80-0.90
Ki_0.90_0.80 <- round(((Simdat$p50_0.90-Simdat$p50_0.80)/Simdat$sigma)[1], 2)
# interval Ki(%) 0.85-0.95
Ki_0.95_0.85 <- round(((Simdat$p50_0.95-Simdat$p50_0.85)/Simdat$sigma)[1], 2)
# interval Ki(%) 0.90-0.999
Ki_0.999_0.90 <- round(((Simdat$p50_0.999-Simdat$p50_0.90)/Simdat$sigma)[1], 2)

# generate interval information for Figure 2 (text on the right in Figure 1A)
p_2A.2 <- ggplot() +
  annotate("text", x = 1, y = 5.1,
           label = "bold('Intervals of ') * bold(K[i])",
           parse = TRUE,
           size = text.size, hjust = 0, size.unit = "pt") +
  annotate("text", x = 1, y = 4.9,
           label = "bold('(% and probit)')",
           parse = TRUE,
           size = text.size, hjust = 0, size.unit = "pt") +

  annotate("text", x = 2, y = 5.1,
           label = "bold('Variability in ') * bold(p[50])",
           parse = TRUE,
           size = text.size, hjust = 0, size.unit = "pt") +
  annotate("text", x = 2, y = 4.9,
           label = "bold('(fraction of ') * sigma * bold(')')",
           parse = TRUE,
           size = text.size, hjust = 0, size.unit = "pt") +

  annotate("text", x = 1, y = 4.1, label = "75 - 85%",
           size = text.size, hjust = 0, size.unit = "pt") +
  annotate("text", x = 1, y = 3.9, label = paste(round(qnorm(0.75),2),"-", round(qnorm(0.85),2)),
           size = text.size, hjust = 0, size.unit = "pt") +
  annotate("text", x = 2, y = 4, label = paste(Ki_0.85_0.75),
           size = text.size, hjust = 0, size.unit = "pt") +

  annotate("text", x = 1, y = 3.6, label = "80 - 90%",
           size = text.size, hjust = 0, size.unit = "pt") +
  annotate("text", x = 1, y = 3.4, label = paste(round(qnorm(0.8),2),"-", round(qnorm(0.9),2)),
           size = text.size, hjust = 0, size.unit = "pt") +
  annotate("text", x = 2, y = 3.5, label = paste(Ki_0.90_0.80),
           size = text.size, hjust = 0, size.unit = "pt") +

  annotate("text", x = 1, y = 3.1, label = "85 - 95%",
           size = text.size, hjust = 0, size.unit = "pt") +
  annotate("text", x = 1, y = 2.9, label = paste(round(qnorm(0.85),2),"-", round(qnorm(0.95),2)),
           size = text.size, hjust = 0, size.unit = "pt") +
  annotate("text", x = 2, y = 3, label = paste(Ki_0.95_0.85),
           size = text.size, hjust = 0, size.unit = "pt") +
```

```
  annotate("text", x = 1, y = 2.6, label = "90 - 99.9%",
           size = text.size, hjust = 0, size.unit = "pt") +
  annotate("text", x = 1, y = 2.4, label = paste(round(qnorm(0.9),2),"-", round(qnorm(0.999),2)),
           size = text.size, hjust = 0, size.unit = "pt") +
  annotate("text", x = 2, y = 2.5, label = paste(Ki_0.999_0.90),
           size = text.size, hjust = 0, size.unit = "pt") +

  xlim(1,2.5) +
  ylim(0,5.2) +
  theme_void() +
  theme(plot.margin= margin(0,0,0,0))

p_2A.2 # show plot
```

**Intervals of K_i (% and probit)**	**Variability in p_{50} (fraction of σ)**
75 – 85%<br>0.67 – 1.04	0.36
80 – 90%<br>0.84 – 1.28	0.44
85 – 95%<br>1.04 – 1.64	0.61
90 – 99.9%<br>1.28 – 3.09	1.81

Illustrate the noise in p50 when Ki(%) restricted

Ki(%) restricted to 85-95%

```
# simulate 500 seed lots with random sigma-values (same as in (2) but
# initial viability restricted to range between 0.9 and 0.8)
lot_number <- 500 # number of random seed lots
viability <- runif(lot_number, min = 0.85, max = 0.95) # random initial viabilities for each
                                                        # seed lot varying between
```

DOI 10.1002/aps3.70031

```
                                                       # min (0.85) and max (0.95)
sigma <- runif(lot_number, min = 1, max = 50) # random values of sigma (in days) for each
                                              # seed lot  varying between
                                              # min (1 day) and max (50 days)
Simdat1 <- data.frame( # dataframe for simulated 500 random seed lots with
  viability = viability, # generated initial viabilities
  sigma = sigma) # and sigma
Simdat1$ID <- row(Simdat)[,1] # assign IDs to simulated species

# calculate p50 for simulated species
Simdat1 <- Simdat1 %>%
  mutate(p50 = qnorm(viability) * sigma)

p_2B <- ggplot(data = Simdat1, aes(x = sigma, y = p50, color = viability)) +
  geom_point(shape = 19, alpha = 0.6, size = point.size) +
  create_gradient(min(Simdat1$viability), max(Simdat1$viability)) +
  labs(x = "Sigma", y = expression(p[50] * " (days)")) +
  ggtitle(expression(K[i] ~"(%) = 85 - 95%")) +
  xlim(0,50) +
  ylim(0,150) +
  theme_classic() +
  theme(
    axis.title.x = element_text(size = lab.size),
    axis.title.y = element_text(size = lab.size),
    axis.text.x = element_text(size = text.size),
    axis.text.y = element_text(size = text.size),
    plot.margin = margin(c(0,0,0,0)),
    plot.title = element_text(size = lab.size))

p_2B # show plot
```

DOI 10.1002/aps3.70031

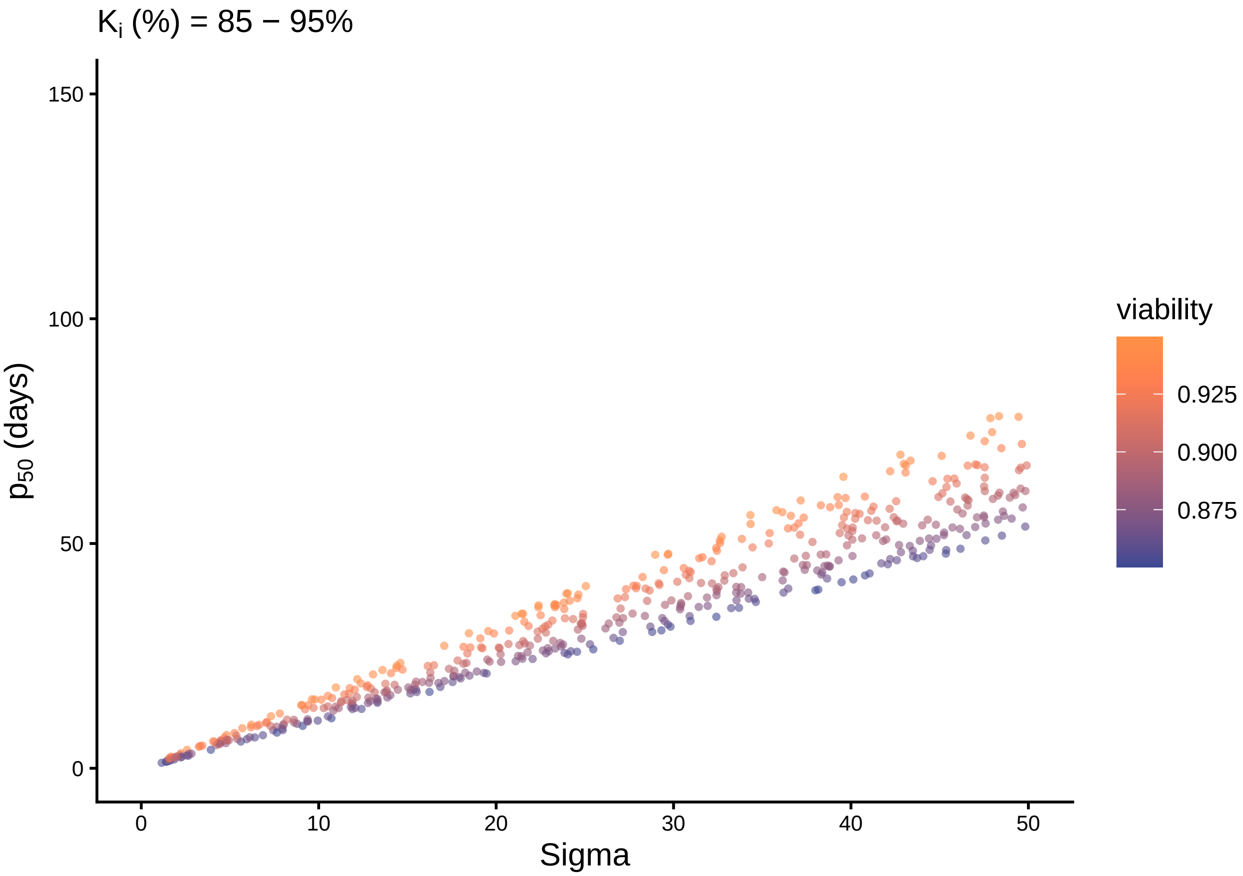

Ki(%) restricted to 90 to 99.9%

```
# simulate 500 seed lots with random sigma-values (same as in (2) but
# initial viability restricted to range between 0.999 and 0.90)
lot_number <- 500 # number of random seed lots
viability <- runif(lot_number, min = 0.90, max = 0.999) # random initial viabilities for each
                                                        # seed lot varying between
                                                        # min (0.90) and max (0.999)
sigma <- runif(lot_number, min = 1, max = 50) # random values of sigma (in days) for each
                                              # seed lot  varying between
                                              # min (1 day) and max (50 days)
Simdat2 <- data.frame( # dataframe for simulated 300 random seed lots with
  viability = viability, # generated initial viabilities
  sigma = sigma) # and sigma
Simdat2$ID <- row(Simdat)[,1] # assign IDs to simulated species

# calculate p50 for simulated species with original initial viability
Simdat2 <- Simdat2 %>%
  mutate(p50 = qnorm(viability) * sigma)  # Original p50 calculation

# Ki(%) limited to 0.9-0.999
p_2C <- ggplot(data = Simdat2, aes(x = sigma, y = p50, color = viability)) +
  geom_point(shape = 19, alpha = 0.6, size = point.size) +
  create_gradient(min(Simdat2$viability), max(Simdat2$viability)) +
```

DOI 10.1002/aps3.70031

```
  # scale_color_gradient2(low = "#374794", mid = "#966070", high = "#F57A50", midpoint =  0.85) +
  labs(x = "Sigma", y = expression(p[50] * " (days)")) +
  ggtitle(expression(K[i] ~"(%) = 90 - 99.9%")) +
  xlim(0,50) +
  ylim(0,150) +
  theme_classic() +
  theme(
    axis.title.x = element_text(size = lab.size),
    axis.title.y = element_text(size = lab.size),
    axis.text.x = element_text(size = text.size),
    axis.text.y = element_text(size = text.size),
    plot.margin = margin(c(0,0,0,0)),
    plot.title = element_text(size = lab.size))

p_2C # show plot
```

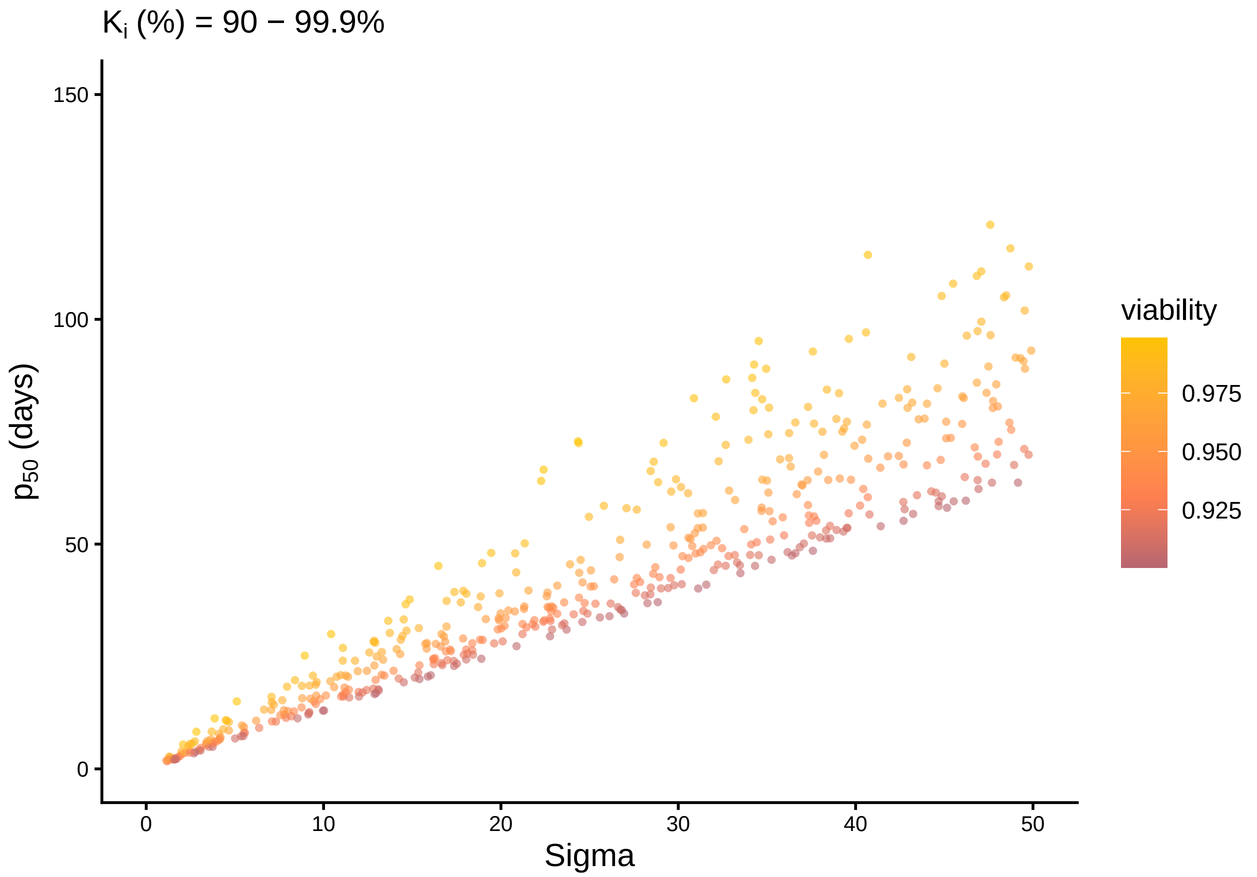

Combine Figure 2

```
# remove legends from p_2B and p_2C
p_2B_nolegend <- p_2B + theme(legend.position = "none")
p_2C_nolegend <- p_2C + theme(legend.position = "none")

p_2 <- plot_grid(
```

DOI 10.1002/aps3.70031

```
  plot_grid(p_2A.1, p_2A.2, ncol = 2,
            labels = c("A", ""), label_size = 12, label_x = 0, label_y = 1,
            rel_widths = c(0.65, 0.35)),  # Adjust the width ratio for the first row
  plot_grid(p_2B_nolegend, p_2C_nolegend, p_legend_gradient, ncol = 3,
            labels = c("B", "C", ""), label_size = 12, label_x = 0, label_y = 1.01,
            rel_widths = c(0.42, 0.42, 0.16)),
  nrow = 2
) + theme(plot.margin = margin(5,15,0,0))

p_2 # show plot
```

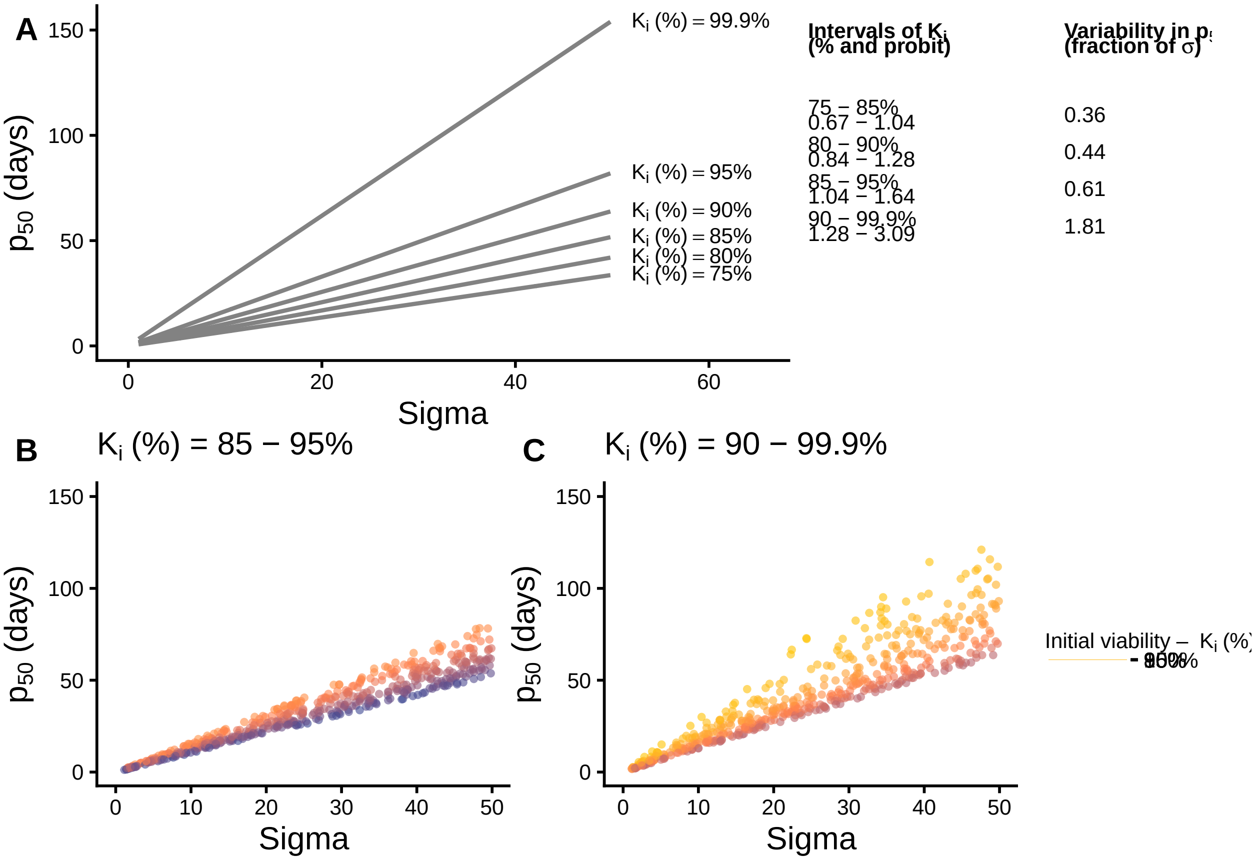


```
# save plot (the saved plots look identical to the Figures in the paper)
# ggsave(file = "your/path/Figure2.pdf", plot = p_2, width = 184, height = 200, units = "mm", dpi=600)
```

3.2 Figure 3: Use sigma instead of p50

- Figure 3 helps to understand the parameter sigma.
- The code is rather long because it contains many design elements.

```
# simulate the extent of the viability loss in one sigma, depending on Ki, sigma is fixed to 1
Ki.percentage <- seq(from=0.999, to=0.023, by= -0.001) # generate initial viabilities
                                                       # between 2.3% and 99.9%
viab_sigma <- pnorm(qnorm(Ki.percentage)-1) # calculate viability after viability loss of one probit
                                            # (equals sigma days in aging)
```

DOI 10.1002/aps3.70031

```r
# qnorm(Ki.percentage): Probit transformation of initial viability,
# qnorm(Ki.percentage)- 1: substract 1 probit
# pnorm(qnorm(Ki.percentage)-1): Back transformation
loss <- Ki.percentage-viab_sigma # calculate viability loss in percentage points
                                 # after sigma days in aging

# Create dataframe for viability loss depending on Ki
Viab_loss <- data.frame(
  Ki.percentage = Ki.percentage,
  viab_sigma = viab_sigma,
  loss = loss)

# Set datapoints for three example initial viabilities to illustrate differences in viability loss
viab_A <- 0.691 # Example point A: 69.1% initial viability
viab_B <- 0.841 # Example point B: 84.1% initial viability
viab_C <- 0.977 # Example point C: 97.7% initial viability

# create dataframe with data for the 3 example points (A, B, C) --> necessary for figure 3
P_3_info <- data.frame(
  point = c("A", "B", "C"),
  viab_start = c(viab_A, viab_B, viab_C)
) %>%
  dplyr::mutate(
    viab_start_rounded = round(viab_start,2),
    viab_end = pnorm(qnorm(viab_start)-1),
    viab_start_time = (-1 * qnorm(viab_start) + 3),
    viab_end_time =  (-1 * qnorm(viab_end) + 3),
    viab_loss = viab_start-viab_end,
    text_y = (viab_start+viab_end)/2
  )

# generate subsets for each example point
P_3A <- P_3_info[P_3_info$point == "A",] # for p_3A
P_3B <- P_3_info[P_3_info$point == "B",] # for p_3B
P_3C <- P_3_info[P_3_info$point == "C",] # for p_3C

# Illustrate viability loss depending on Ki
p_3.1 <- ggplot(data = Viab_loss, aes(x = Ki.percentage, y = loss)) +
  geom_line(colour="#FF7F50") +
  geom_segment(aes(x = Inf, xend = P_3A$viab_start,
                   y = P_3A$viab_loss, xend = P_3A$viab_loss),
               color = "#374794", size = 0.6) +
  geom_segment(aes(x = Inf, xend = P_3B$viab_start,
                   y = P_3B$viab_loss, xend = P_3B$viab_loss),
               color = "#FFC300", size = 0.6) +
  geom_segment(aes(x = Inf, xend = P_3C$viab_start,
                   y = P_3C$viab_loss, xend = P_3C$viab_loss),
               color = "#9B4D96", size = 0.6) +
  geom_point(aes(x=P_3A$viab_start, y=P_3A$viab_loss), shape = 21,
             size = point.size+2.5, colour = "#374794", fill = "#B3C6E7") +
  geom_point(aes(x=P_3B$viab_start, y=P_3B$viab_loss), shape = 21,
             size = point.size+2.5, colour = "#FFC300", fill = "#FFF59D") +
  geom_point(aes(x=P_3C$viab_start, y=P_3C$viab_loss), shape = 21,
```

DOI 10.1002/aps3.70031

```
           size = point.size+2.5, colour = "#9B4D96", fill = "#E1BEE7") +

# Text annotation
  annotate("text",
         x = P_3A$viab_start,
         y = P_3A$viab_loss,
         label = paste0("K[i]~'(%)'==", P_3A$viab_start * 100, "*'%'"),
         parse = TRUE,
         hjust = -0.2,
         size = text.size, size.unit = "pt") +
  annotate("text",
         x = P_3B$viab_start,
         y = P_3B$viab_loss,
         label = paste0("K[i]~'(%)'==", P_3B$viab_start * 100, "*'%'"),
         parse = TRUE,
         hjust = -0.2,
         size = text.size,
         size.unit = "pt", size.unit = "pt") +
  annotate("text",
         x = P_3C$viab_start,
         y = P_3C$viab_loss,
         label = paste0("K[i]~'(%)'==", P_3C$viab_start * 100, "*'%'"),
         parse = TRUE,
         hjust = -0.2,
         size = text.size, size.unit = "pt") +
  labs(x = expression("Initial viability - "~ K[i]~" (%)"),
     y = "Viability loss per one sigma (% points)") +

  scale_x_reverse(labels = scales::percent) +
  scale_y_continuous(limits = c(0,0.4), labels = scales::percent) +
  # ylim(0.2,0.4) +
  theme_classic() +
  theme(
    axis.title.x = element_text(size = lab.size),
    axis.title.y = element_text(size = lab.size),
    axis.text.x = element_text(size = text.size),
    axis.text.y = element_text(size = text.size),
    plot.margin = margin(0,0,0,10))

p_3.1 # show plot
```

DOI 10.1002/aps3.70031

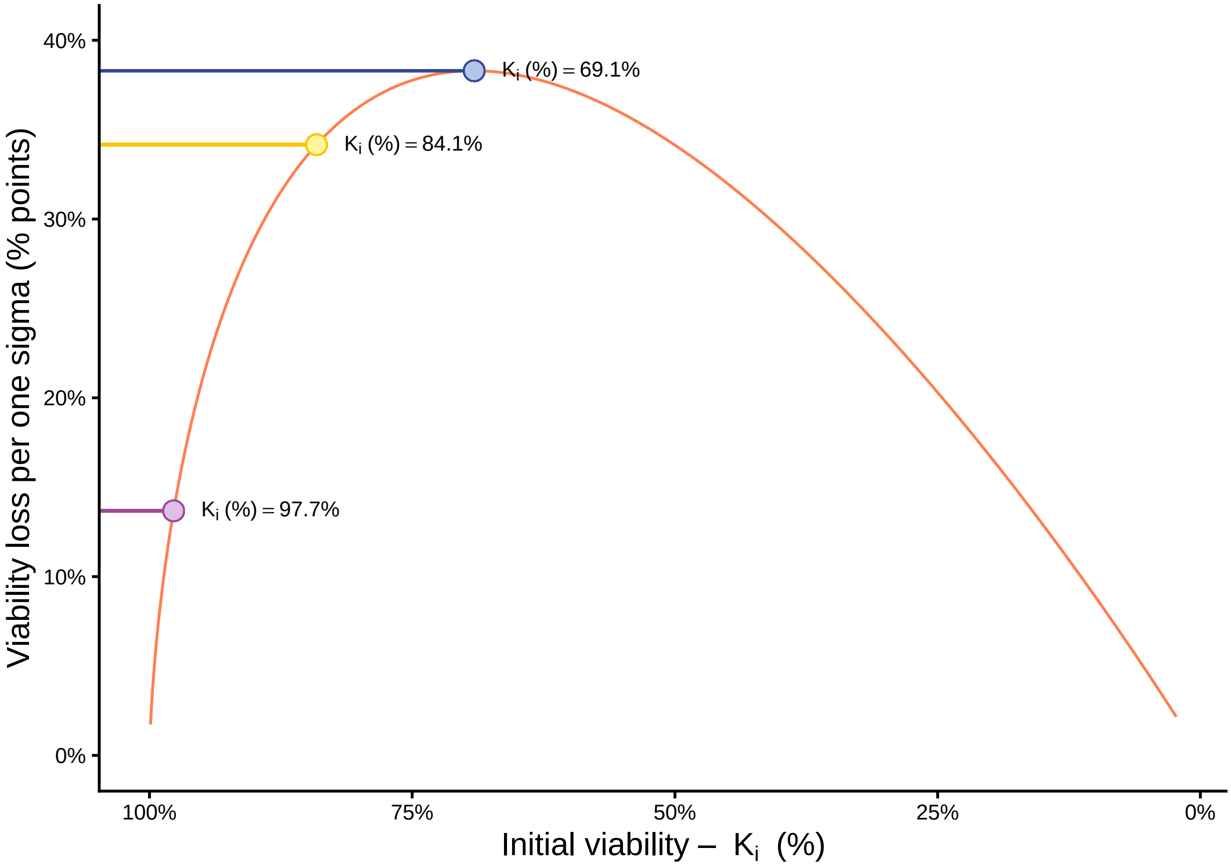

Illustrate viability loss for each of the three example points

Figure 3A

```
p_3A <- p_seed_survival +
  # horizontal line
  geom_segment(data = P_3A,
               aes(x = viab_start_time, xend = viab_end_time,
                   y = viab_end, yend = viab_end),
               color = "grey80", size = 0.6) +
  # Sigma annotation
  annotate("text",
           x = P_3A$viab_start_time + 0.5,
           y = P_3A$viab_end,
           hjust = 0.5,
           vjust = -0.3,
           label = "sigma", size = text.size, size.unit ="pt",
           parse = TRUE
  ) +
  # Plot points from dataframe on seed survival
  geom_line(data = Seed_survival %>%
              dplyr::filter(seed_viability >= P_3A$viab_end & seed_viability <= P_3A$viab_start),
            size = line.size, colour = "#374794") +
  # Arrow
  geom_segment(data = P_3A,
```

DOI 10.1002/aps3.70031

```
               aes(x = viab_start_time, xend = viab_start_time,
                   y = viab_start, yend = viab_end),
               color = "#374794", size = 0.6,
               arrow = arrow(ends = "last", length = unit(0.02, "npc"))) +
  # Starting point viability loss
  geom_point(data = P_3A,
             aes(y = viab_start, x = viab_start_time),
             shape = 21, size = point.size + 2.5, colour = "#374794", fill = "#B3C6E7") +
  # Text: Viability loss
  annotate("text",
           x = P_3A$viab_start_time,
           y = 0.5 * (P_3A$viab_start + P_3A$viab_end),
           hjust = 1.1,
           label = paste0("Viability loss\n= ", round(P_3A$viab_loss, 3)*100, "% points"),
           size = text.size, size.unit ="pt") +
  ylab(expression("Initial viability - "~ K[i]~" (%)"))

p_3A # show plot
```

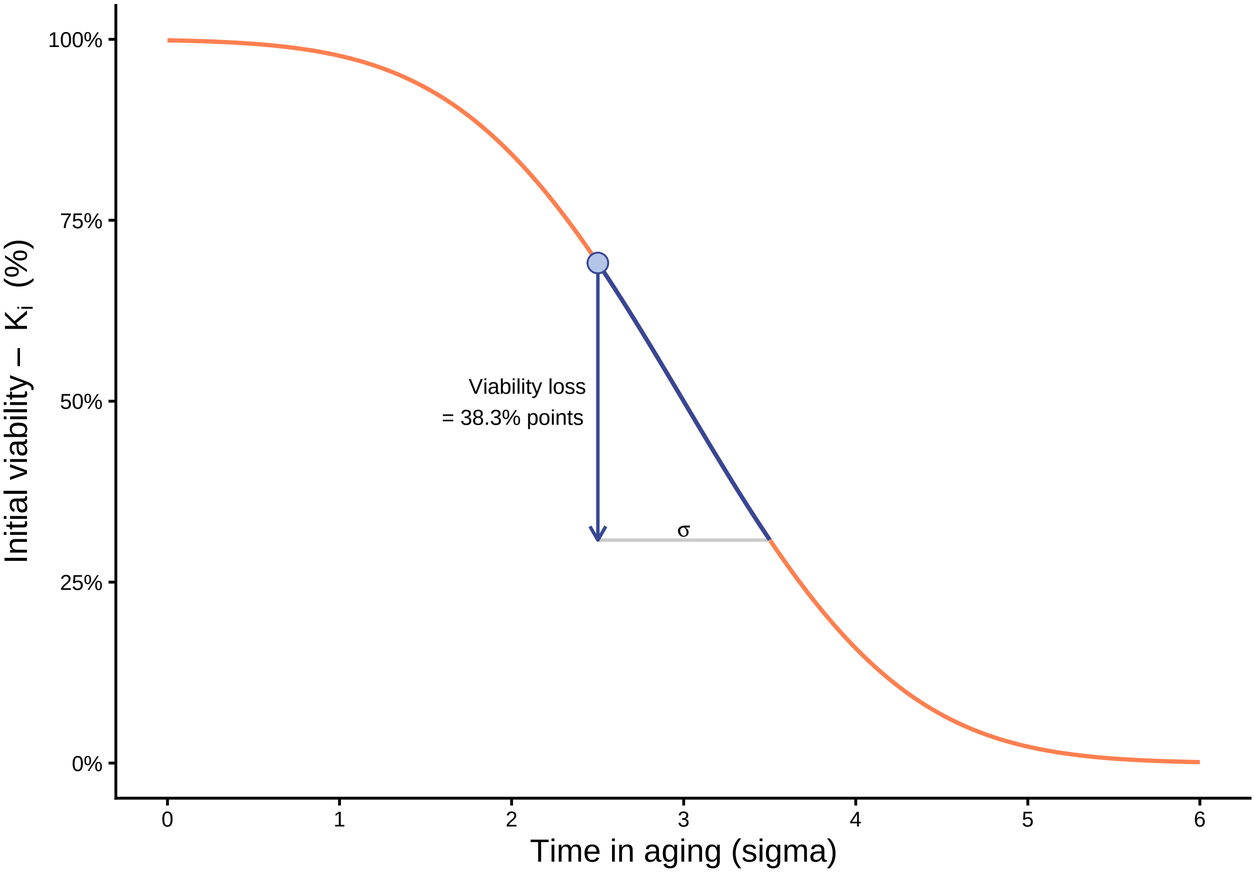

Figure 3 B

```
p_3B <- p_seed_survival +
  # horizontal line
  geom_segment(data = P_3B,
               aes(x = viab_start_time, xend = viab_end_time,
                   y = viab_end, yend = viab_end),
```

DOI 10.1002/aps3.70031

```
                color = "grey80", size = 0.6) +
  # Sigma annotation
  annotate("text",
           x = P_3B$viab_start_time + 0.5,
           y = P_3B$viab_end,
           hjust = 0.5,
           vjust = -0.3,
           label = "sigma", size = text.size, size.unit ="pt",
           parse = TRUE
  ) +
  # Plot points from dataframe on seed survival
  geom_line(data = Seed_survival %>%
               dplyr::filter(seed_viability >= P_3B$viab_end & seed_viability <= P_3B$viab_start),
            size = line.size, colour = "#FFC300") +
  # Arrow
  geom_segment(data = P_3B,
                aes(x = viab_start_time, xend = viab_start_time,
                    y = viab_start, yend = viab_end),
                color = "#FFC300", size = 0.6,
                arrow = arrow(ends = "last", length = unit(0.02, "npc"))) +
  # Starting point viability loss
  geom_point(data = P_3B,
              aes(y = viab_start, x = viab_start_time),
              shape = 21, size = point.size + 2.5, colour = "#FFC300", fill = "#FFF59D") +
  # Text: Viability loss
  annotate("text",
           x = P_3B$viab_start_time,
           y = 0.5 * (P_3B$viab_start + P_3B$viab_end),
           hjust = 1.1,
           label =
             paste0("Viability loss\n= ", round(P_3B$viab_loss, 3)*100, "% points"),
           size = text.size, size.unit ="pt") +
  ylab(expression("Initial viability - "~ K[i]~" (%)"))

p_3B # show plot
```

DOI 10.1002/aps3.70031

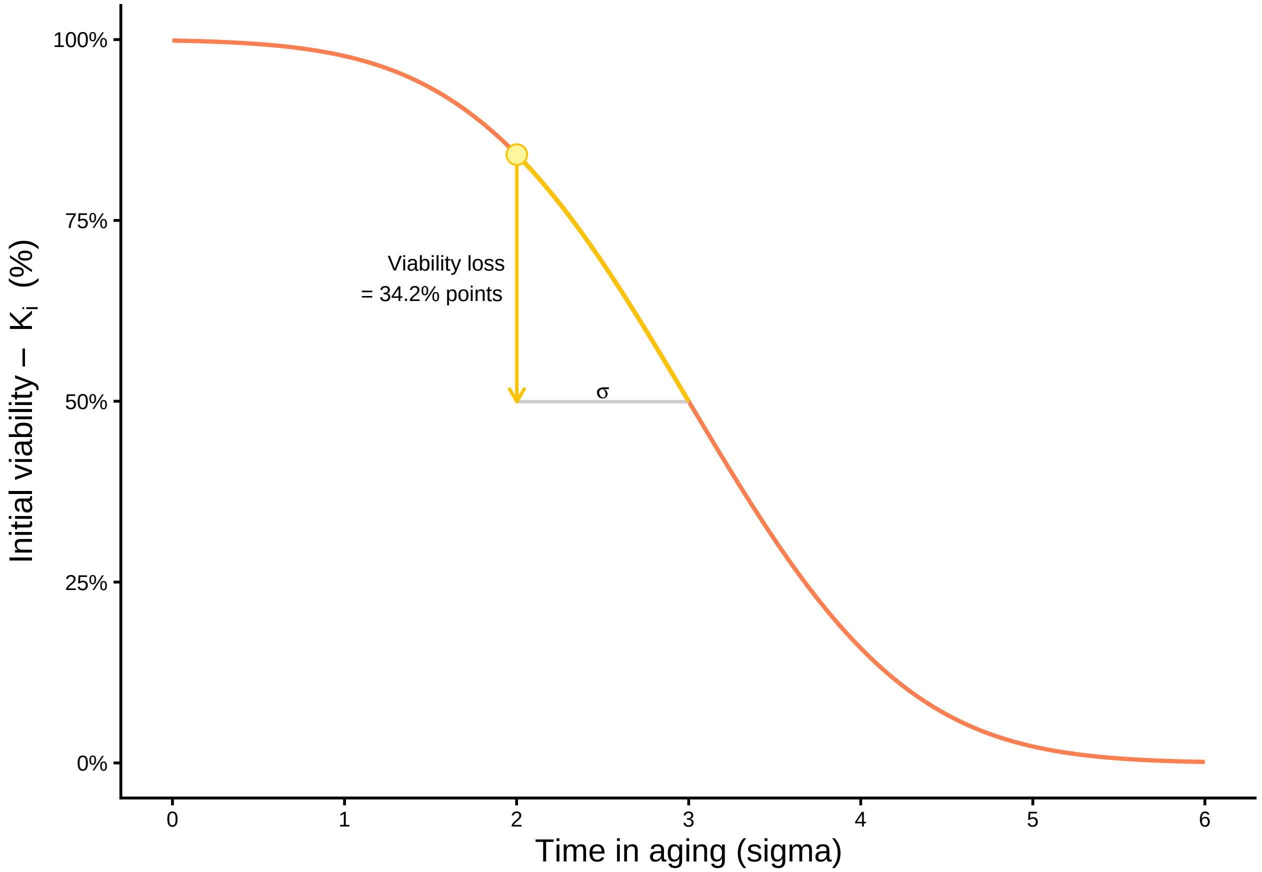

Figure 3C

```
p_3C <- p_seed_survival +
  # horizontal line
  geom_segment(data = P_3C,
               aes(x = viab_start_time, xend = viab_end_time,
                   y = viab_end, yend = viab_end),
               color = "grey80", size = 0.6) +
  # Sigma annotation
  annotate("text",
           x = P_3C$viab_start_time + 0.5,
           y = P_3C$viab_end,
           hjust = 0.5,
           vjust = -0.3,
           label = "sigma", size = text.size, size.unit ="pt",
           parse = TRUE
  ) +
  # Plot points from dataframe on seed survival
  geom_line(data = Seed_survival %>%
              dplyr::filter(seed_viability >= P_3C$viab_end & seed_viability <= P_3C$viab_start),
             size = line.size, colour = "#9B4D96") +
  # Arrow
  geom_segment(data = P_3C,
               aes(x = viab_start_time, xend = viab_start_time,
                   y = viab_start, yend = viab_end),
               color = "#9B4D96", size = 0.6,
```

DOI 10.1002/aps3.70031

```
                arrow = arrow(ends = "last", length = unit(0.02, "npc"))) +
  # Starting point viability loss
  geom_point(data = P_3C,
             aes(y = viab_start, x = viab_start_time),
             shape = 21, size = point.size + 2.5, colour = "#9B4D96", fill = "#E1BEE7") +
  # Text: Viability loss
  annotate("text",
           x = P_3C$viab_start_time,
           y = 0.5 * (P_3C$viab_start + P_3C$viab_end),
           hjust = -0.45,
           label =
             paste0("Viability loss = ", round(P_3C$viab_loss, 3)*100, "% points"),
           size = text.size, size.unit ="pt") +
  ylab(expression("Initial viability - "~ K[i]~" (%)"))

p_3C # show plot
```

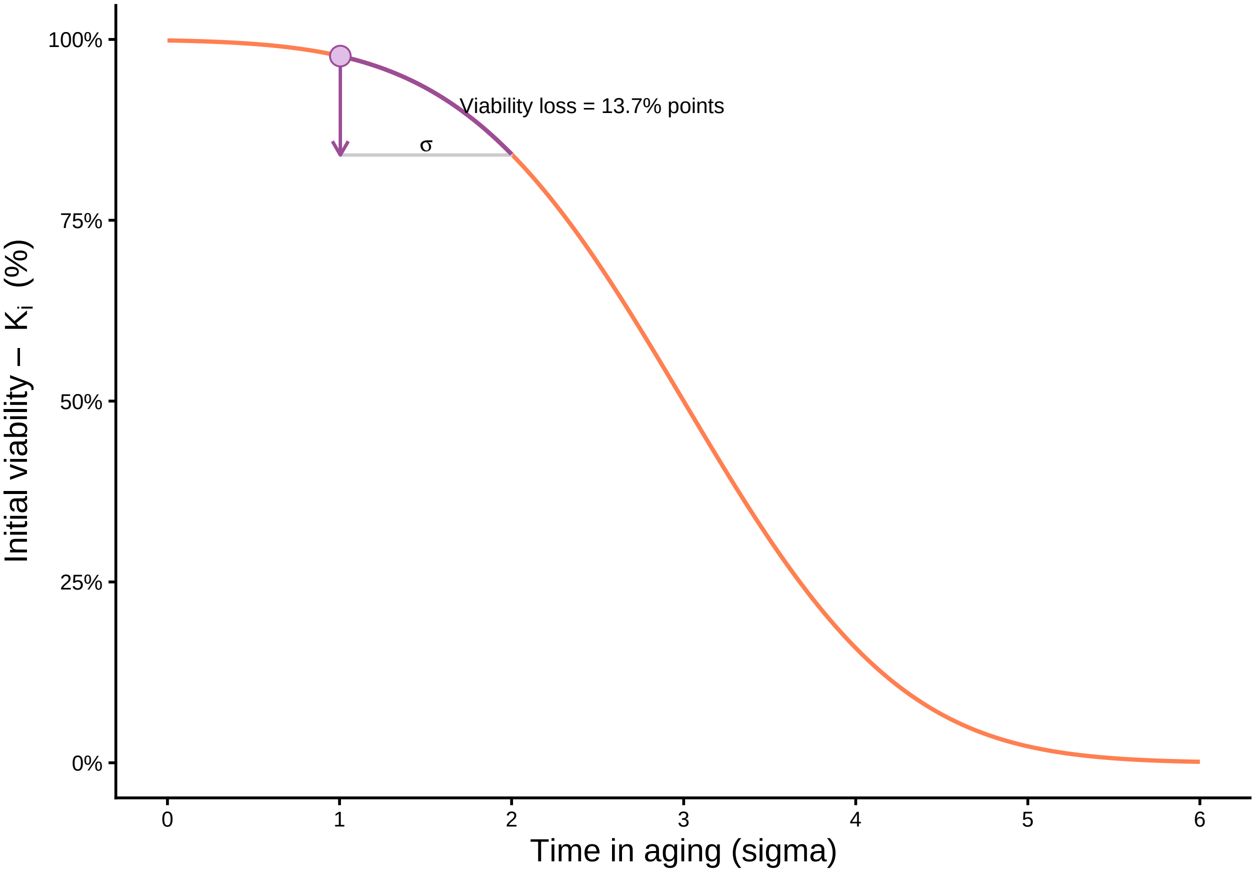

Combine plots for Figure 3

```
p_3 <- plot_grid(
  plot_grid(p_3A, p_3B, p_3C, ncol = 1, nrow = 3,
            labels = c("A", "B", "C"),
            label_size = 12, label_x = -0.01, label_y = 1),
  #           rel_heights = c(1, 1, 1)),
  p_3.1, ncol = 2,
  labels = c("", "D"), label_size = 12, label_x = 0, label_y = 1,
```

DOI 10.1002/aps3.70031

```
  rel_widths = c(0.5, 0.5)
)

p_3 # show plot
```

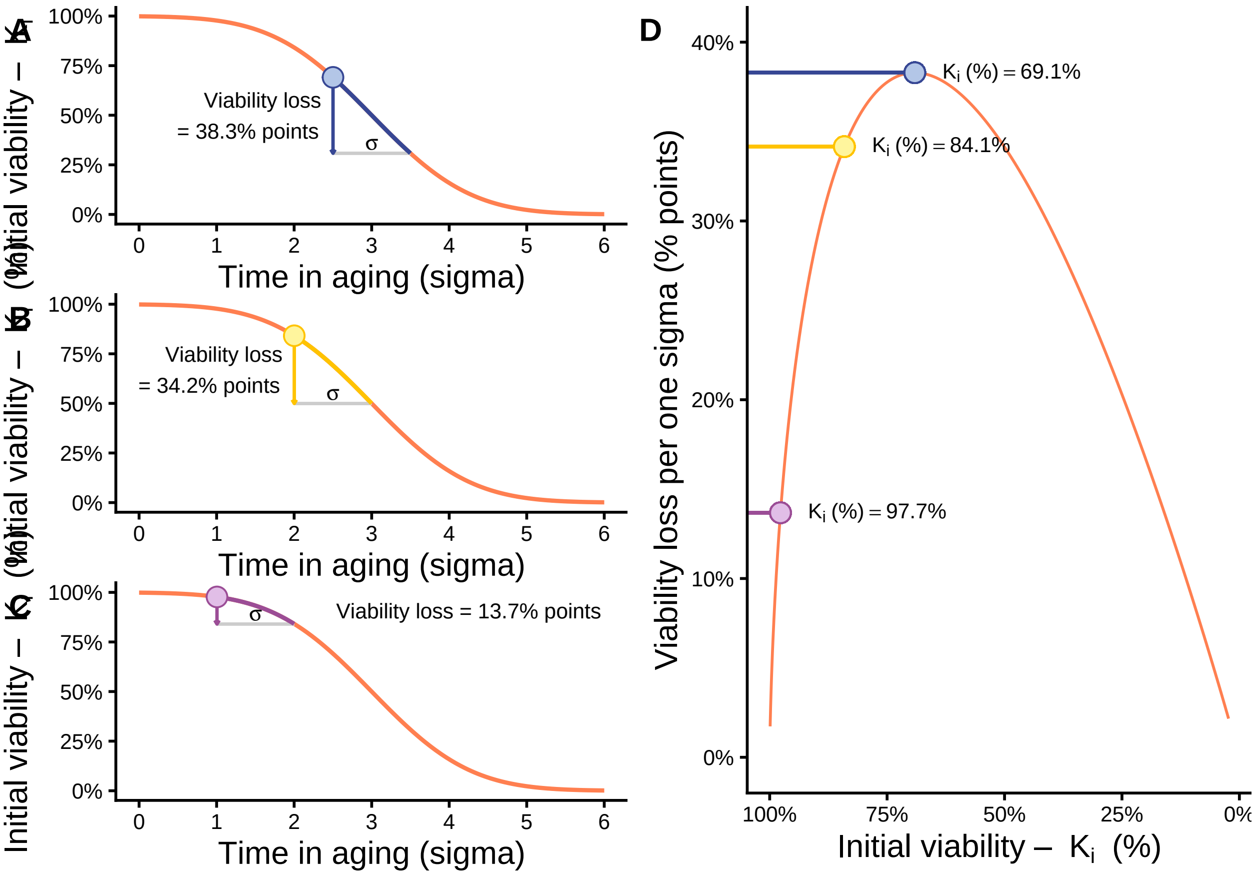


```
# save plot (the saved plots look identical to the Figures in the paper)
# ggsave(file= "your/path/Figure3.pdf", plot = p_3, width = 184, height = 200, units = "mm", dpi=600)
```

3.3 Standardized p50

see Figure 2A
